# Region- and time-dependent gene regulation in the amygdala and anterior cingulate cortex of a PTSD-like mouse model

**DOI:** 10.1186/s13041-019-0449-0

**Published:** 2019-03-28

**Authors:** Mikiei Tanaka, Hongyun Li, Xijun Zhang, Jatinder Singh, Clifton Dalgard, Matthew Wilkerson, Yumin Zhang

**Affiliations:** 1 Department of Anatomy, Physiology and Genetics, Uniformed Services University of Health Sciences, 4301 Jones Bridge Rd, Bethesda, MD 20814 USA; 2 Collaborative Health Initiative Research Program (CHIRP), Uniformed Services University of Health Sciences, 4301 Jones Bridge Rd, Bethesda, MD 20814 USA

**Keywords:** Synaptic remodeling, Neuroendocrine, Long-term regulation, Anxiety, Avoidance, Hyperarousal, Sleep, RNA-seq, Amygdala, Anterior cingulate cortex

## Abstract

Posttraumatic stress disorder is developed by exposure to a threatening and/or a horrifying event and characterized by the presence of anxiety, hyperarousal, avoidance, and sleep abnormality for a prolonged period of time. To elucidate the potential molecular mechanisms, we constructed a mouse model by electric foot shock followed by situational reminders and performed transcriptome analysis in brain tissues. The stressed mice acquired anxiety-like behavior after 2 weeks and exaggerated startle response after 4 weeks. Avoidance latency and freezing behavior were sustained up to 5 weeks post stress and abnormal static behavior was observed during the sleep period. RNA sequencing was performed in two of the emotional regulatory regions, anterior cingulate cortex and amygdala, at 2 and 5 weeks post stress. More than 1000 differentially expressed genes were identified at 2 weeks in both regions. The number of the regulated genes remained constant in amygdala at 5 weeks post stress, whereas those in anterior cingulate cortex were plummeted. Although synaptic remodeling and endocrine system were the most enriched signaling pathways in both anterior cingulate cortex and amygdala, the individual gene expression profile was regulated in a region- and time-dependent manner. In addition, several genes associated with PTSD involved in Hypothalamic-Pituitary-Adrenal axis were differentially regulated. These findings suggested that global gene expression profile was dynamically regulated in accordance with the disease development stage, and therefore targeting the distinct signaling molecules in different region and development stage might be critical for effective treatment to PTSD.

**Electronic supplementary material:**

The online version of this article (10.1186/s13041-019-0449-0) contains supplementary material, which is available to authorized users.

## Introduction

Post-traumatic stress disorder (PTSD) is a psychiatric disorder defined by profound disturbances in cognition, emotion, behavior and physiological function in response to a life threatening or a horrifying traumatic event. The symptoms of PTSD are characterized by three long-lasting symptomatic clusters (DSM-5): (1) re-experiencing (intrusive thoughts, recurrent nightmares, flashbacks and reactivity to reminders of the trauma); (2) avoidance and emotional numbness (avoiding stimuli associated with trauma and anhedonia); and (3) hyperarousal (hypervigilance, exaggerated startle response and sleep disturbance). PTSD patients may also exhibit impaired cognition, working memory, and reduced social activities. Another characteristic of PTSD is that those symptoms emerge long after traumatic events in some patients, suggesting that disease can develop after an extended asymptomatic period.

Anxiety disorders including PTSD encompass heterogeneous conditions that are associated with abnormalities in brain regions controlling fear response such as the medial prefrontal cortex, amygdala (AMY), hippocampus, and nucleus accumbens [1, 2]. Changes in activity and functional connectivity of these brain regions lead to the development and maintenance of anxiety disorders [3–5]. AMY is a critical brain region involved in the detection of threat, fear learning and expression, and heightened emotional memory. The exaggerated activity in AMY following traumatic events is correlated with the severity of symptoms in PTSD patients. AMY is modulated by the cortical influence from medial prefrontal cortex. The altered activity in the anterior cingulate cortex (ACC), a subregion of medial prefrontal cortex, has been demonstrated in PTSD using functional MRI [6]. With extensive connection to limbic structures including AMY, ACC is thought to be the direct top-down regulator of PTSD susceptibility through modulating AMY activation to threatening stimuli [7, 8]. In particular, dorsal ACC is thought to facilitate the expression of conditioned fear and increased neuronal activity in PTSD during fear conditioning [2, 9]. The volume of ACC was consistently found to decrease in PTSD, however some studies have revealed hyperactivation of the ACC rather than hypoactivation [10]. Therefore, it is important to investigate the functional activity of ACC in relation to AMY hyperactivation in PTSD.

Recent genomic profiling and neuroimaging studies for human PTSD have uncovered several candidate genes for the pathogenesis, however detailed investigation on gene regulation and the underlying mechanisms are still limited [11–13]. Molecular and cellular encoding of traumatic events and behavioral responses can be reflected by the changes of synaptic plasticity. Specifically, signaling molecules associated with synaptic transmission and plasticity in connecting to AMY are implicated as primary substrates for fear learning and memory. Alteration in glutamatergic and GABAergic neurotransmission contributes to abnormal behavioral responses [14]. In addition, downstream signaling components, such as MAPK, cAMP and calcium mediated signaling molecules are involved in synaptic plasticity [15]. The alteration in several neuromodulatory systems such as endocannabinoid signaling, Wnt signaling and ErbB signaling can affect fear memory [16] and anxiety-like behavior by synaptic remodeling in medial prefrontal cortex [17] and AMY [18, 19].

Hypothalamic-pituitary-adrenal (HPA) axis plays a pivotal role in stress response upon exposure to emotional or physical stressors, and its feedback loop mediated by cortisol is altered in PTSD [20, 21]. In fact, human genetic studies identified several PTSD-associated genes are involved in HPA axis and cortisol, for instance FKBP5 and corticotropin releasing hormone receptors [22]. Oxytocin and thyroid hormone signaling pathways are modulated by cortisol and the imbalance may cause emotional lability, impatience, anxiety and sleep disturbances [23, 24].

As PTSD diagnostic criteria are not met at least 1 month after traumatic event according to DSM-5, sustained psychological abnormality is one of the prominent features. However, the molecular mechanisms underlying these psychological changes are not well-addressed. In this study we employed genome-wide unbiased gene analysis using RNA-seq to characterize the regulation of differentially expressed genes in a stress induced PTSD-like mouse model and found that several signaling pathways involved in synaptic remodeling and endocrine systems, as well as PTSD-associated genes were regulated in a region- and time-dependent manner.

## Materials and methods

### Animals

**Fig. 1:**
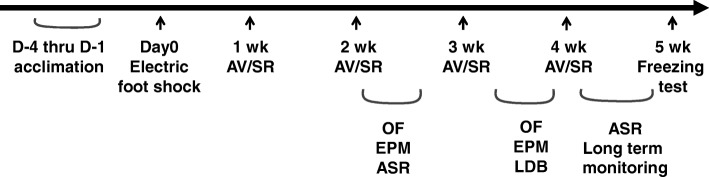
Timeline of PTSD-like model construction and behavior tests. C57BL/6J mice at 8–10 weeks of age were subjected to acclimation followed by electric foot shock. The control mice underwent the same acclimation procedure without foot shock. Avoidance test and situational reminder (AV/SR) were weekly performed, and the freezing test was done at 5 weeks post stress (PS). Open field test (OF) and elevated plus maze test (EPM) were performed at 2 weeks and 4 weeks PS. Light/dark box test (LDB) was performed around 4 weeks PS. Acoustic startle response test (ASR) was performed around 2 weeks and 4 weeks PS, and locomotive activity test were carried out from 4 weeks to 5 weeks PS

Male, 8 to 10 weeks old C57BL/6J mice were purchased from Jackson Laboratories (Bar Harbor, ME). Animals were maintained under standard conditions of controlled temperature (23 °C) and housed in a 12 h light/dark cycle (light on at 6 AM) with access to water and food ad libitum. They were group housed with 3 to 5 mice per cage and habituated to the vivarium for at least 3 days. Experimental procedures were carried out in accordance with NIH guidelines and approved by the Uniformed Services University Animal Care and Use Committee. One hundred twenty-four mice in total were used for performing various behavioral tests (Fig. 1) and molecular analyses.

### Foot shock induction

Animal model for PTSD-like symptoms was constructed by foot shock induced fear conditioning and re-exposure to the shock chamber as previously described [25, 26]. Briefly, trauma procedure consisted of a single exposure to an inescapable electric foot shock (Day 0) followed by re-exposure to the shock chamber every week until 4 weeks (Fig. 1). Mice received 15 s handling for 3 consecutive days before given foot shock. On the shock day, each animal was placed in the light compartment. After a 2 min adaptation period, the door to the dark shock apparatus made of plexiglass (17 cm × 17 cm × 25 cm) was open to let the animal entering the shock chamber. After another 5 min adaptation in the closed chamber, the animal received inescapable foot shock (1.5 mA × 2 s with 2 min interval for 8 times). The control group received the same procedures without stress. Animals were removed from the apparatus 1 min after the last shock.

### Behavioral test

All the behavioral tests were performed during the light on phase from 8 AM to 3 PM. Mice were given one test a day to avoid the potential interference from the other tests (Fig. 1). Animal cages were moved to a testing room at least 1 h prior to each test. After completion of the test session, the behavioral apparatus and chamber were cleansed with 70% ethanol, and then dried by hand-fan completely.

#### Avoidance latency/situational reminder and freezing tests

For measuring avoidance latency, animals were placed in a compartment adjacent to a foot shock chamber. After 30 s acclimation, the door was open and the time spent before entering foot shock chamber was measured within a period of 2 min. Test was terminated once mouse entered, and the mouse was immediately removed from the shock chamber to a new cage. For measuring freezing time, animals in 5 weeks post stress were placed inside chamber with the same context, and mouse behavior was monitored for 5 min with the digital video system. The data was analyzed by ANY-maze software (Stoelting Co, Wood Dale, IL) to determine freezing time and the number of episodes.

#### Open field test

Each mouse was placed in the center of a cubic chamber (40 cm × 40 cm × 35 cm) with a black Plexiglas wall under dim light in the testing room. The position and movement of animals were monitored for 10 min by video camera installed above the chamber. Data analyses were carried out by ANY maze software to determine the moving velocity, total travel distance, the time spent in the center zone, the entry number to the center zone. Center zone was assigned in a 20 cm × 20 cm square area in the center of arena.

#### Elevated plus maze test

The apparatus consisted of four arms (34 cm × 5 cm width), with two arms open and two closed by gray walls (15 cm height) arranged in the opposite side of the same type. The platform was located 50 cm above the floor of the testing room illuminated and four arms were connected in the center platform (5 cm × 5 cm), where animal was placed facing to a closed arm. The position and movement of animal was monitored for 5 min by video camera. An entry was defined if more than half of the animal’s full body entering the open arm. The time spent in the open arms and number of visits to the open arms were analyzed using ANY-maze.

#### Light/dark box test

The light–dark box was made of white and black opaque Plexiglas (40 × 20 × 30 cm light chamber, 23 × 20 × 30 cm dark chamber). The chambers were connected by a 6 × 6 cm door in the middle of the wall separating the two chambers. Animals were placed in the middle of the dark chamber with the door closed. Video monitoring was started when the door was open and continued for 10 min to measure transition number into the lit area, retention time in lit, and travel distance inside lit using ANY-maze software.

#### Acoustic startle test

Startle response was measured using ventilated startle chambers (SR-LAB system, San Diego, CA). Sound levels within each chamber were measured using a sound-level meter to ensure consistent presentation. Animals were placed in a Plexiglas cylinder (∅4.7 cm × 10 cm long) inside a chamber (29 cm × 29 cm × 29 cm). The cylinder resting on a platform was connected to a piezoelectric accelerometer to monitor movement. Each test session was started with a 3 min acclimation period to background white noise of 70 dB, followed by 10 times acoustic stimulus (120 dB × 20 ms) with a 1 min interval. Animals were returned to home cage immediately after test session. Data analyses were carried out by SD software (SR-LAB system) and summarized with Microsoft Excel. The latency to first peak, maximum amplitude, and average startle amplitude from 1 ms to 100 ms were analyzed.

#### Home-cage immobility test

Video, magnet, photobeam, or infrared sensor system has been used to measure home cage activity [27]. This system has been applied for monitoring sleep-wake behavior instead of polysomnography [28, 29]. Group housed animals were separated individually into a cage (20 cm × 38 cm) and nourished with water gel and food ad libitum for 3 days. One day prior to the test, the cages were placed on a test platform before 6 PM to adapt the environment. The room light was turned off at 6 PM and turned on at 6 AM the following day, in accordance with the previous light-dark cycle. Locomotive activity was monitored immediately after 6 AM with the video camera system and continued for 11.5 h. Data was analyzed numerically by ANY maze software to determine the immobile period. Locomotive activity during the entire test period was dissected with a 2 min interval to assess immobile period which showed no locomotion within each 2 min-section. Total travel distance and total immobile time were measured by ANY maze. Short immobile episode was determined as the number of immobile periods of 1 or 2 sequential immobile sections.

### RNA extraction

Mice were sacrificed after deep anesthetization from 10 AM to 2 PM. Brains were immediately fresh frozen on dry ice and stored at − 80 °C. Coronal section of the brain at 300 μm was cut at − 20 °C. Three adjacent brain sections of bilateral amygdala (AMY) (Bregma − 0.94 mm to − 1.84 mm) and those of bilateral anterior cingulate cortex (ACC) (Bregma + 1.34 mm to + 0.44 mm) [30] were collected by punching with 1 mm-diameter brain punch based on the method described by Palkovits [31]. Total RNAs from AMY and ACC were isolated by Trizol (Invitrogen, Carlsbad, CA) and purified with the RNeasy Mini Kit (Qiagen, Germantown, MD). The RNA concentration was measured by Qubit 3.0 Fluorometer (Invitrogen).

### RNA-seq

Library preparation for RNA-seq was performed by TrueSeq stranded mRNA sample preparation kit (Illumina, San Diego, CA) starting from 600 ng of total RNA. Sequence data were generated by Illumina 3000 and NextSeq platforms. We converted raw BCL files to FASTQ files and performed multiplexing using Illumina bcl2fastq2 software (version 2.17). Sequencing quality control was done by Fastqc program. We aligned the resulting FASTQ files to mm 10 mouse reference genome by STAR aligner. Quantification to read counts, FPKM and TPM was done by RSEM program. With raw read counts matrix as input, we identified genes that were differentially expressed by DESeq2 R package, using unpaired two-class significance analysis and a false discovery rate threshold of 0.05. Gene expression visualization was plotted by heatmap R package. KEGG Pathway and GO analysis were performed by the Database for Annotation, Visualization and Integrated Discovery (DAVID) v6.8 [32] or “DOSE” R Bioconductor package [33].

### Statistics

The GraphPad Prism 7 (GraphPad Software Inc., San Diego, CA) was used for statistical analysis. The weekly avoidance latency test was analyzed by two-way ANOVA followed by Bonferroni’s multiple comparison test. The statistical comparison between the two groups was determined by Welch’s unpaired two tailed t-test. All data are expressed as mean ± SEM. Statistical significance was set at *p* < 0.05. *, **, and *** depicting *p* < 0.05, *p* < 0.01, and *p* < 0.001, respectively.

## Results

### Sustained avoidance and fear memory in foot shock stressed mice

**Fig. 2:**
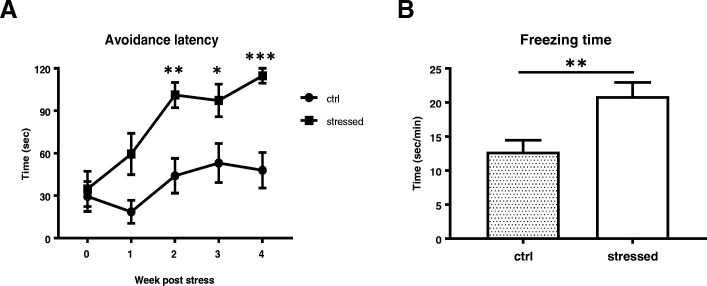
Latency to avoidance and the freezing behavior test. The latency to enter the foot shock chamber was tested weekly and the stressed mice had a prolonged latency compared to the control mice (**a**). At 5 weeks PS, the freezing behavior inside the chamber was monitored for 5 min and analyzed by ANY maze software (**b**). Data are represented as mean ± S.E.M. (*n* = 14/group), Two-way ANOVA with Bonferroni’s test in (**a**) and unpaired two-tailed t-test in (**b**) were performed

For assessment of avoidance behavior, latency till moving into the same foot shock chamber from the light compartment was measured every week. The latency of stressed mice was longer than that of control mice starting from the first week and sustained for at least 4 weeks (F_4,104_ = 2.782, *p* = 0.0305) (Fig. 2a). Notably, almost all stressed mice did not move out from light compartment to the stressed chamber except for the first trial (Fig. 2a). When placed inside the same shock chamber at 5 weeks after stress, the stressed mice possessed a longer freezing time compared to the control mice (*p* = 0.0038) (Fig. 2b), although the frequency of freezing episode was not significantly altered.

### Anxiety-like behavior emerged at 2 weeks post stress

**Fig. 3:**
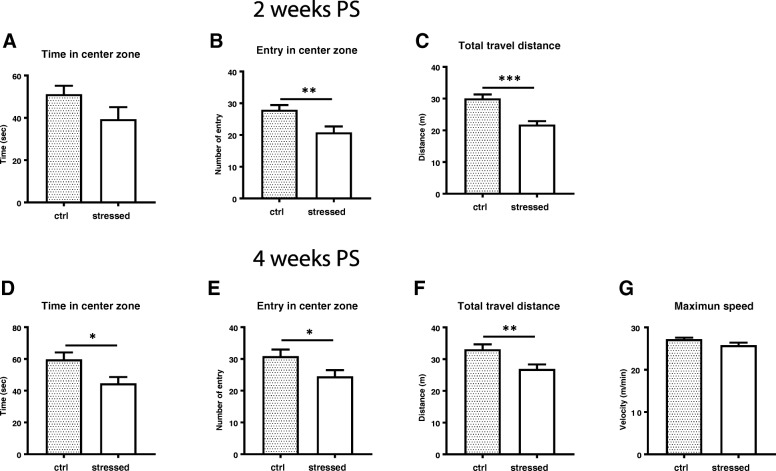
Anxiety-like behavior assessed by Open field test. Mice were placed in a cubic square under dim light and allowed to move freely for 10 min. Mice movement related to anxiety was assessed based on the time in center zone (**a** and **d**), entry number to center zone (**b** and **e**), total travel distance (**c** and **f**), and maximum speed (**g**). Panels **a**, **b**, and **c** show the results at 2 weeks PS, panels **d**-**g** show those at 4 weeks PS. Unpaired two-tailed t-test was performed. Data are represented as mean ± S.E.M. (*n* = 24/group at 2 weeks PS, *n* = 34/group at 4 weeks PS)

**Fig. 4:**
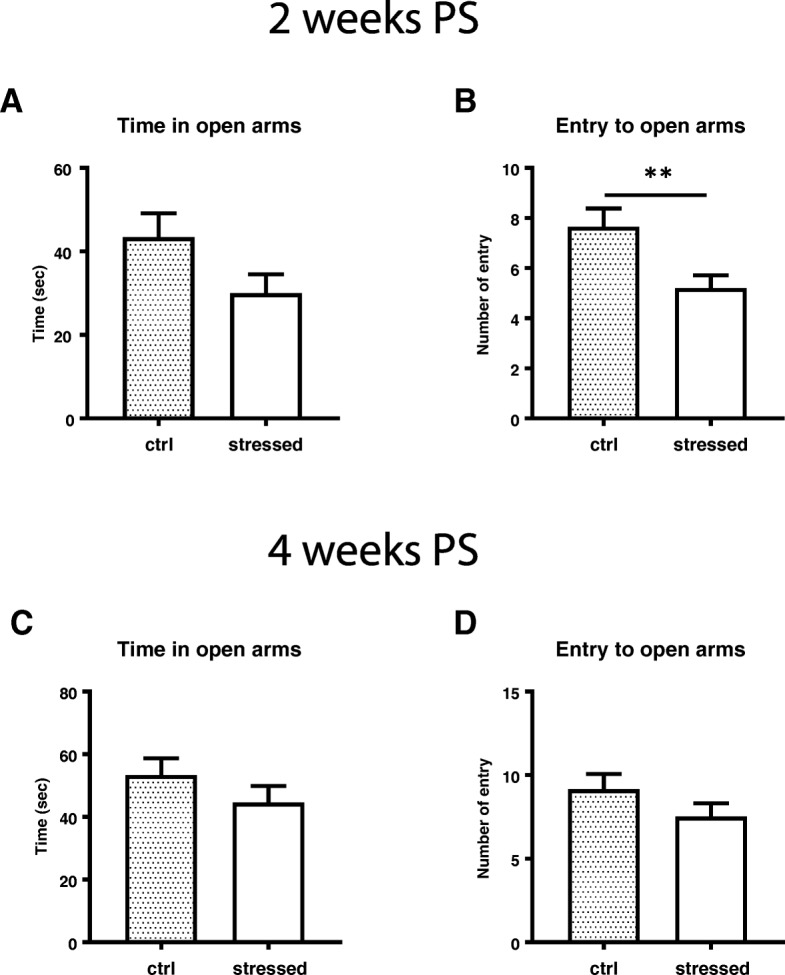
Anxiety-like behavior assessment by elevated plus maze test, mice were placed in a plus maze apparatus and allowed to move freely for 5 min. Time in open arms (**a** and **c**) and entry number to open arms (**b** and **d**) were measured at 2 weeks PS (**a** and **b**) or 4 weeks PS (**c** and **d**). Unpaired two-tailed t-test was performed. Data are represented as mean ± S.E.M. (*n* = 24/group at 2 weeks PS, *n* = 34/group at 4 weeks PS)

**Fig. 5:**
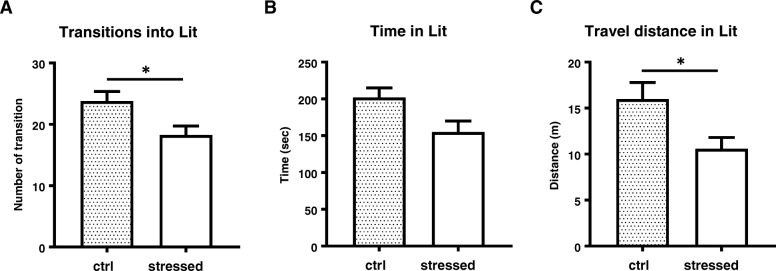
Anxiety-like behavior assessment by light/dark box test. Mice at 4 weeks PS were freely moved in an arena divided by light and dark areas connected with a small door for 10 min. Animal movement in terms of transition frequency (**a**), retention time in lit area (**b**), and travel distance in lit are (**c**) were analyzed. Unpaired two-tailed t-test was performed. Data are represented as mean ± S.E.M. (*n* = 16/group)

As anxiety is a typical behavioral feature in PTSD, we examined if stressed mice acquired and sustained anxiety using several behavioral tests. Open field test was performed in a black walled squire space for 10 min. At 2 weeks PS, the stressed mice showed decreased retention time in the center zone and entry number to the center zone (*p* = 0.0056) (Fig. 3a, b). Total travel distance was significantly shorter in the stressed mice than control mice (*p* = 0.0006) (Fig. 3c). At 4 weeks PS, the stressed mice showed significant reduction in retention time in the center zone (Fig. 3d, *p* = 0.0126), entry number to the center zone (Fig. 3e, *p* = 0.0304), and total travel distance (Fig. 3f, *p* = 0.0047). Shorter travel distance was unlikely caused by motor deficit of the stressed mice, since maximum running speed was not impaired (Fig. 3g). In the elevated plus maze test, the stressed mice spent less time in open arms than control mice at 2 weeks PS, despite the significant difference was not reached (Fig. 4a). The entry number to open arms was significantly lower in the stressed mice (*p* = 0.0095) (Fig. 4b). When examined at 4 week PS, there were no significant differences in both time spent and entry number in open arms between the stressed mice and control mice (Fig. 4c and d). To further examine the anxiogenic behavior at these time points, we also performed a light/dark box test. It was shown that the number of transitions to light area and travel distance in light area during 10 min test period were significantly reduced in the stressed group than the control group (Fig. 5a and c). Although time spent in light area was not significantly different (Fig. 5b), the stressed mice preferred dark protected area and less explored in an unprotected area compare to control mice at 4 weeks PS. Overall, these tests demonstrated that the stressed animals started to show anxiety-like behavior at 2 weeks PS and continued till 4 weeks PS.

### Hyperarousal symptoms in stressed mice

**Fig. 6:**
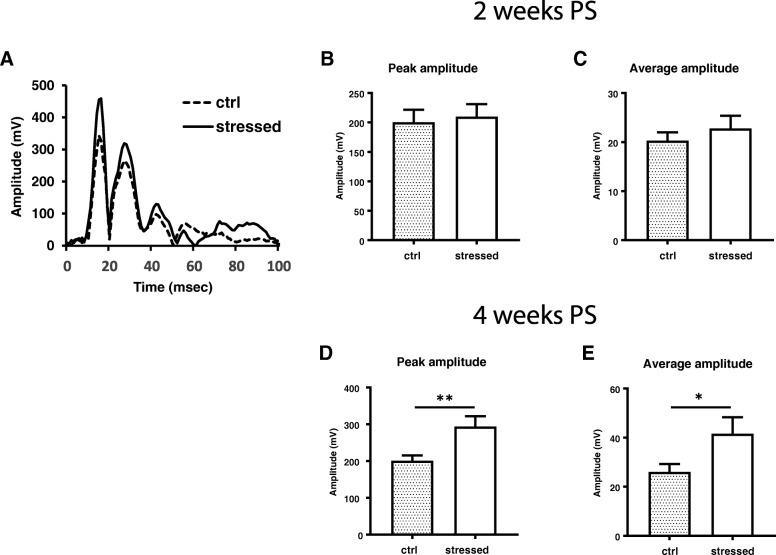
Acoustic startle response test for control and stressed mice. Mice were acclimated in the chamber under 70 dB background noise for 3 min, followed by start acoustic stimulus (120 dB × 20 msec) 10 times in 1 min interval. Representative startle response profiles were shown in (**a**) for control as broken line and for stressed mice as solid line. The average of 10 trials of peak response amplitude was measured at 2 weeks PS (**b**) and 4 weeks PS (**d**). The average amplitude from 1 ms to 100 ms was measured at 2 weeks PS (**c**) and 4 weeks PS (**e**). Unpaired two-tailed t-test was performed. Data are represented as mean ± S.E.M. (*n* = 24/group at 2 weeks PS, *n* = 34/group at 4 weeks PS)

Acoustic startle response against 120 dB stimulus repeated for ten times were monitored by a motion sensor system, and the maximum peak amplitude, latency to the first peak, and average amplitude were measured. As shown in Fig. 6a, both control and stressed mice showed a similar pattern to startle response. The average amplitude of the maximum peak out of 10 trials was not different between control and stressed mice at 2 weeks PS. The average amplitude from 1 ms to 100 ms was also similar despite a slight increase observed in the stressed mice (Fig. 6c). However, at 4 weeks PS the peak amplitude was significantly higher in the stressed mice (*p* = 0.0048) (Fig. 6d), and the average amplitude was also greater in stressed mice than control mice (*p* = 0.0440) (Fig. 6e). There was no difference in startle response latency between the stressed and control mice at either test period. These results suggested that startle response was exaggerated in stressed animals at relatively later time point.

### Increased frequency of short immobile episodes during the sleep period

**Fig. 7:**
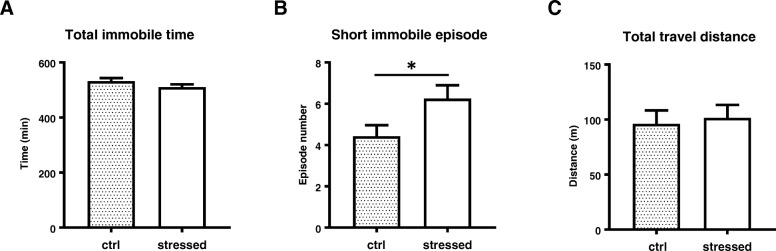
Characterization of immobile pattern during inactive phase. Locomotive activity in home-cage was monitored by video camera during inactive phase for 11.5 h and analyzed using ANY-maze software. Total immobile time was shown in (**a**), and number of short immobile episode (immobile duration between 2 min and 4 min) was shown in (**b**), and total travel distance was shown in (**c**). Data are represented as mean ± S.E.M. (*n* = 28/group). Unpaired two-tailed t-test was performed

Sleep disturbance is a common feature and impacts the pathophysiology of PTSD and other psychiatric diseases [34]. We characterized the sleep-wake behavior of stressed mice with long-term video monitoring in their home cages. There was no significant difference between control and stressed mice in total immobile time during the entire inactive phase (Fig. 7a). However, the number of short immobile episodes was significantly larger in the stressed mice than the control mice (*p* = 0.0353) (Fig. 7b), suggesting that the sleep/wake cycle was disturbed in stressed mice. The total travel distance showed no significant difference between the control and stressed mice, indicating the locomotive activity was not impaired in stressed animals (Fig. 7c).

### Differentially expressed genes in ACC and AMY

We performed genome wide RNA expression analysis in the anterior cingulate cortex (ACC), which is involved in the modulation of emotional and fear encoding and consolidation. Of the 3233 differentially expressed genes (DEGs) in ACC at 2 weeks post stress (PS), 1335 were upregulated, and 1898 were downregulated (Additional file 1: Figure S1A). However, at 5 weeks PS the numbers plunged to 43 (Additional file 1: Figure S1B) and only 16 of them were overlapped between these time points (Additional file 1: Figure S1C). In AMY 2 weeks PS, there were 1063 DEGs, in which 873 genes were predominantly down-regulated (Additional file 2: Figure S2A). Different from ACC, there were a substantial number of DEGs in 5 weeks PS (Additional file 2: Figure S2B). Of more than 1000 DEGs, approximately 9% (94 DEGs) were overlapped between 2 weeks and 5 weeks PS as shown in the Venn diagram (Additional file 2: Figure S2C).

### Regulation of PTSD associated genes in the mouse model

Several human genomic studies have been conducted recently to identify candidate genes for PTSD patients [35–38]. We found that ten genes associated with PTSD were regulated in either AMY or ACC (Table 1). Several of these genes were involved in HPA axis and glucocorticoid regulation, which include *Crhr1* and *2, Fkbp5, Rora, Sgk1,* and *Stat5b*. Other genes including *Adcy8, Cacna1c, Klhl1*, and *Shank1* were involved in synapse plasticity and regulation. Gene expression regulation in our animal model was correlated, at least to some extent, with that identified in different brain areas or blood samples from patients with PTSD.

**Table 1:** Regulation of PTSD-associated genes

Gene symbol	Gene name	region	time	Regulation	Regulation in PTSD	Reference
Adcy8	adenylate cyclase 8	AMY	5	Up		[38]
Cacna1c	calcium voltage-gated channel subunit alpha1C	ACC	2	Dw		[72]
Crhr1	Corticotropin releasing hormone receptor 1	ACC	2	Up		[73]
Crhr2	Corticotropin releasing hormone receptor 2	ACC	2	Dw		[74]
Fkbp5	FK506 binding protein 5	ACC	2	Dw	Up (orbito-frontal cortex) or Dw (subgenual PFC)	[22, 75, 76]
Klhl1	Kelch like family member 1	AMY	2	Dw		[36]
Oxtr	Oxytocin receptor	AMY	2	Up		[77]
Rora	RAR related orphan receptor A	ACC	2	Up		[78]
Sgk1	serum/glucocorticoid regulated kinase 1	AMY	2	Dw	Dw (PFC)	[49, 79]
Shank1	SH3 and Multiple Ankyrin repeat doamin1	ACC	2	Dw	Up (subgenual PFC)	[75]
Stat5b	signal transducer and activator of transcription 5B	AMY	5	Dw	Dw (blood)	[50]
Tbc1d2	TBC1 Domain Family Member 2	AMY	2	Dw		[35]
Tbc1d2	TBC1 Domain Family Member 3	ACC	2	Up		[35]

### Differentially expressed genes in AMY

Those DEGs in AMY were applied for Gene Ontology (GO) analysis using DAVID to determine enriched functional annotation in terms of molecular functions (Additional file 3: Table S1) or biological processes (Additional file 4: Table S2). First, to characterize the regulation of global mRNA expression, we searched for the GO terms related to transcriptional regulation and found comparable numbers of DEGs in the Transcription and Negative regulation of transcription between 2 weeks and 5 weeks PS, whereas the Positive regulation of transcription was enriched only in 5 weeks PS (Additional file 5: Figure S3). Of note, the Covalent chromatin modification was enriched only in 2 weeks PS. Consistently, there were four more enrichments related to the chromatin modifications: unmethylated CpG binding, histone deacetylase binding, histone H3-K4 methylation and histone H4-K5 acetylation.

There were several gene regulations in terms of cellular alteration in the central nervous system (CNS) (Additional file 6: Figure S4). Nervous system development was enriched both in 2 weeks and 5 weeks PS, while neuron projection development was only seen in 2 weeks PS. Neurogenesis and Neural precursor cell proliferation were enriched in 5 weeks PS. GOs related to neuron differentiation were enriched both in 2 weeks PS and in 5 weeks PS. For non-neuronal cell differentiation, Oligodendrocyte differentiation and Epithelial cell differentiation were enriched in 2 and 5 weeks PS, respectively.

### Gene regulation in AMY

**Fig. 8:**
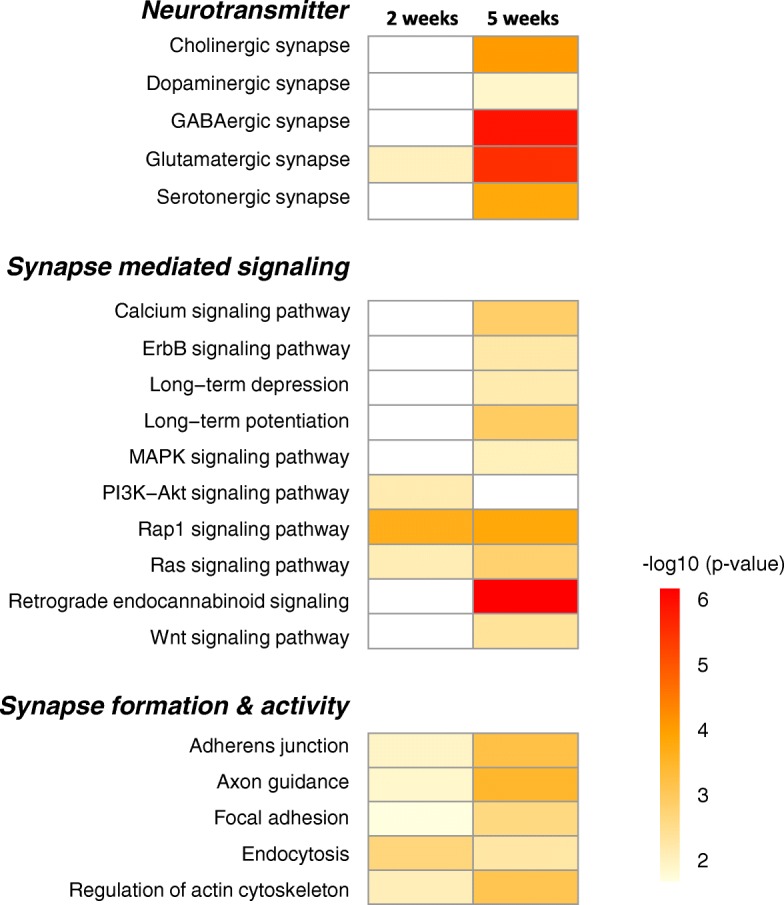
Enriched KEGG pathways related to synapse remodeling in AMY. Enriched pathways were categorized into 3 functions, involved in neurotransmitter (upper panel), synapse mediated signaling (middle panel), and synapse formation and activity (lower panel). Each pathway in the two time points was represented as heatmap with color index based on *p*-value

A number of KEGG pathways were involved in synaptic remodeling (Fig. 8) from the enriched pathway analyses (Additional file 7: Table S3). Pathways for neurotransmitters including acetylcholine, dopamine, GABA, glutamate, and serotonin were enriched in 5 weeks PS. We found gene regulation of glutamatergic kainite receptor subunits, *Grik3, 4* and *5*, metabotropic receptor subunits, *Grm4* and *7*. In GABAergic synapse, type A GABAergic receptor subunits (*Gabrg1, Gabrg3,* and *Gabrq*) and GABA transporter (*Slc38a1*) were regulated after 5 weeks. Moreover, acetylcholine esterase, *Ache,* and muscarinic cholinergic receptor, *Chrm3*, dopamine receptor1, *Drd1*, and serotonergic receptors, *Htr1b, 1d, 2c,* and *7* were regulated.

In terms of synapse mediated signaling, LTP and LTD pathways and several downstream signaling pathways crucial for synapse plasticity such as MAPK, Ras, and PI3K/Akt were enriched. Ras signaling pathways including *Mras, Kras*, as well as Ras GTPase regulatory factors, and Rap1 signaling pathways including Rap1 GTPase regulatory factors were overlapped in both 2 weeks and 5 weeks PS. And the key molecules in calcium mediated signaling, calmodulin (*Calm2*), CAM kinase (*Camk2a*), and CREB binding protein (*Crebbp*) which are involved in LTP and LTD were also altered. The key components of Wnt signaling, four receptor genes and three ligands including β-catenin, were regulated. Notably, the retrograde endocannabinoid signaling was highly regulated at 5 weeks PS. Moreover, pathways related to synaptic formation and activity, including Adherens junction, Axon guidance, Focal adhesion, Endocytosis, and Regulation of actin cytoskeleton pathways were enriched.

To further characterize the gene regulation of synapse remodeling, the pathways overlapped in 2 weeks and 5 weeks PSs were characterized by individual DEGs. Most of DEGs were regulated either after 2 weeks or 5 weeks PS, but there were a few DEGs overlapped in both time periods, particularly involved in the Endocytosis pathway (Table 2). These results suggest that individual DEG was differentially regulated at 2 weeks and 5 weeks PS. Consistent with the differential regulation of synapse remodeling genes in AMY, DEGs related to transcription showed only a few overlapped genes between 2 and 5 weeks PS, suggesting a diverse transcriptional regulation.

**Table 2:** DEGs in four representative overlapped pathways and transcription in AMY

	2 weeks only	2 and 5 weeks	5 weeks only
Glutamatergic synapse	*ADCY3, ADCY6, GLS2, GLUL, GRIK3, GRIK4, GRM4, ITPR3, SHANK2*	*PLCB4, MAPK1*	*ADCY5, ADCY7, GNG4, GRM7, PRKACB, SLC17A6, SLC1A6, SLC38A1, ADCY1, ADCY8, DLGAP1, GNG5, GRIK5, HOMER2, MAPK3, PRKCG, SLC17A7*
Ras signaling	*FGFR2, FGFR3, FGF16, MET, HGF, FOXO4, RALGDS, BRAP, KSR2, RASGRP3, PLCG1, VEGFA, RASA3, ABL1, KSR1*	*MAPK1, KITL, FGF22*	*PDGFB, MRAS, ELK1, FGF13, PRKCG, PTPN11, IGF1R, HTR7, RASSF1, MAPK3, RASGRP2, RIN1, PRKACB, PLA2G3, FGF1, GNG4, GNG5, CALM1*
Axon guidance	*PLXNA1, LIMK1, PLXNB1, PLXNB3, MET, EPHB2, SEMA5B, UNC5B, SEMA3G, SEMA4D, ABL1*	*EPHB1, SEMA3F, MAPK1*	*NTNG2, CXCL12, SLIT3, PTK2, SEMA6D, UNC5A, EPHA8, NCK1, MAPK3, ROBO3, NFATC2, SRGAP1, SEMA4A*
Endocytosis	*FGFR2, ARFGAP2, FGFR3, ERBB3, KIF5A, MET, ASAP2, ARPC5, LDLRAP1, RAB7, AMPH, RAB11FIP4, RAB11FIP5, ZFYVE27, ARRB1, FOLR1, GIT2, H2-T24, SMURF1, AGAP1, IQSEC1, MVB12B*	*IQSEC3*	*PARD3, RET, USP8, PSD3, PIP5K1C, SMAD3, VPS37C, CYTH2, CAPZB, SRC, CHMP2B, IGF1R, CHMP1A, FAM21, HSPA2, NTRK1, ACAP2, SPG20, PARD6G, EPN1, IQSEC2*
Regulation of actin cytoskeleton	*FGFR2, FGFR3, SSH1, LIMK1, PPP1R12B, WASF2, FGF16, GNA12, ITGB4, ARPC5, VAV2, PFN4, ITGA7, FGD3*	*FGF22, MAPK1, WASF1*	*PDGFB, ACTN4, MRAS, MYLK3, ITGA11, PIP5K1C, FGF13, ITGA4, SRC, INSRR, ITGA9, PTK2, CHRM3, ITGAV, ITGA8, ARAF, MAPK3, FGF1*
Transcription GO:0006351	*ARNT2, FOXO3, FOXO4, ZIC2, ZGPAT, ZFP932, OLIG3, PPP1R1B, CREB3L2, ZFP687, ZFP276, SATB1, MED12, ZFP629, ZHX3, RRP8, TRERF1, ZFP592, ASCC2, KDM2A, ARRB1, FLII, VGLL4, MYBBP1A, TSHZ2, LITAF, SETD1B, SOX4, TRRAP, SOX8, XAB2, CASZ1, NKX3–1, MTERF2, TCF25, HIP1, MAFG, DNMT3A, IKZF4, TAF3, BHLHE22, SMAD7, TAF8, CREBBP, FOXP2, SREBF2, SAFB2, HDAC4, NOTCH1, SMARCC2, DNMT1, HIVEP1, KAT6B, ADAR, FOXK1, HR, LBH, SUPT5, KDM5C, FOXJ2, SNAPC1, SNAPC4, TLE3, SPEN, MXD4, EYA4, HIPK2, ERN1, MYRF, CUX2, KMT2D, KMT2A, KMT2B, GON4L, MLF1, MINA, ZKSCAN17, SAFB, BCL11B, PRDM11, POU2F2, POU3F3, GATAD2B, POU3F2, BCL6, POU3F1, GTF3C1, CHD5, ZFP526, BRD2, L3MBTL1, PHF12, AFF1, SAP30BP, NKAPL, MEF2D, RPAP1, HNRNPUL1, THRAP3, KDM4B, MAMLD1, ZFP536*	*AKIRIN2, ATMIN, ARID3B, FOXQ1, JADE1, IL33, MXD1, MAPK1, ZFP740*	*MEF2C, THRA, PRR13, STAT5B, CBX4, MED25, ASCC1, CBX2, CBX6, RCBTB1, ZFP92, EPC1, SMARCD3, RARB, ZFP503, MAP 2 K6, MAGEL2, BEND6, ZHX2, SIX3, RXRG, SKOR1, GTF2IRD1, MAPK3, ZFP697, TFAP2C, CAMTA2, HMGB3, ELK1, MEIS1, TAL1, VDR, TRP53BP1, MEIS2, HEXIM2, DRAP1, LHX2, TCF4, SLC30A9, NAT14, RFX5, KLF13, TAF7, PTPN14, NR4A2, SMAD3, NR4A1, FEZF2, ZFHX4, PHF1, DLX5, EBF1, HOPX, HABP4, RFX3, ZFHX3, CREBRF, PAX6, ZEB1, ZFP641, KCNIP3, ARHGAP22, DYNLL1, YAP1, HSF4, BHLHE41, THRSP, INO80C, ZCCHC12, TBL1XR1, KHDRBS3, SSBP2, POLR1D, RUNX1T1, GTF2H5, GTF2B, NRIP2, MYT1L, CCND1, EYA1, CHMP1A, PIH1D1, HIPK2, CUX1, PEG3, THAP7, SEC14L2, ZBTB18, TSPYL2, ZFP664, GBX1, PER3, ZFP521, NFATC2, CHD6, ETV4, MAF, ZFP57, MNAT1, DBP, DACH2, HEYL, NEUROD2, NEUROD1, SP8, TBL1X, SP9, PBX3*

**Fig. 9:**
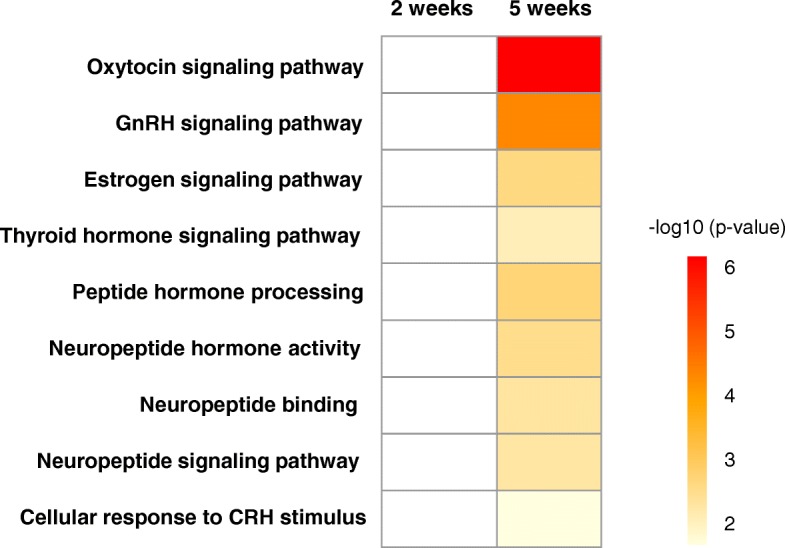
Enriched KEGG pathways related to hormone and neuroendocrine system in AMY. Nine pathways that were enriched based on DEGs in AMY were represented as heatmap with color index based on *p*-value

GO analysis and pathway analysis revealed that several GOs and pathways were involved in hormone and neuroendocrine, particularly in 5 weeks PS but not at 2 weeks PS (Fig. 9), among them are Oxytocin, Thyroid hormone, Gonadotropin releasing hormone, and Estrogen signaling pathways. Oxytocin (*Oxt*) was regulated at both 2 and 5 weeks PS, while oxytocin receptor (*Oxtr*) was only in 2 weeks PS. Thyroid hormone receptors and transporter (*Thra, Rxrg*^,^ and *Slc16a2*) were regulated in 5 weeks PS.

### Gene regulation in ACC

**Fig. 10:**
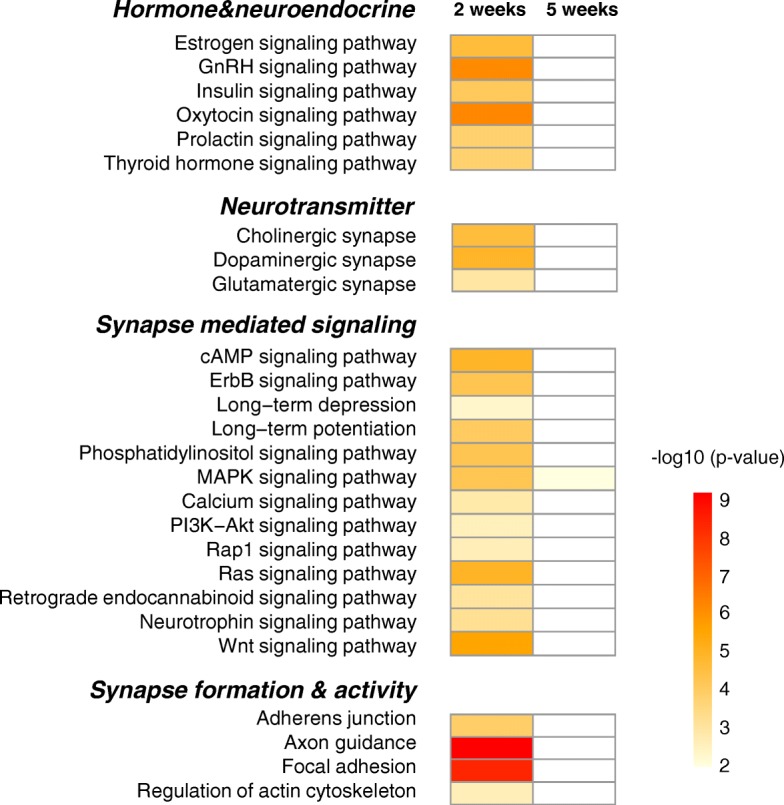
KEGG pathways enriched in ACC 2 weeks and 5 weeks PS. Substantial number of DEGs in ACC showed enrichment in hormone and neuroendocrine system or synapse remodeling. The enriched pathways from the DEGs were represented in heatmaps, and these pathways included hormone & neuroendocrine (upper panel), neurotransmitter (upper middle panel), synapse mediated signaling (lower middle panel) and synapse formation & activity (lower panel). Color index represents the level of significance (*p*-values)

There were substantial number of GOs and pathways enriched in 2 weeks PS. As seen in AMY, we found several GOs and KEGG pathways related to hormone and neuroendocrine system and synapse remodeling. Figure 10 shows enriched KEGG pathways including Estrogen signaling, GnRH signaling, Insulin signaling, Oxytocin signaling, Prolactin signaling, and Thyroid hormone signaling. Substantial number of KEGG pathways were also involved in synapse remodeling. MAPK signaling pathway was the only pathway seen in 5 weeks PS among them. Thus, synapse remodeling and hormone and neuroendocrine system were the mainly regulated pathways in 2 weeks PS, and they were mostly terminated in 5 weeks PS.

### Gene regulation related to PTSD-like behavior in AMY and ACC

**Fig. 11:**
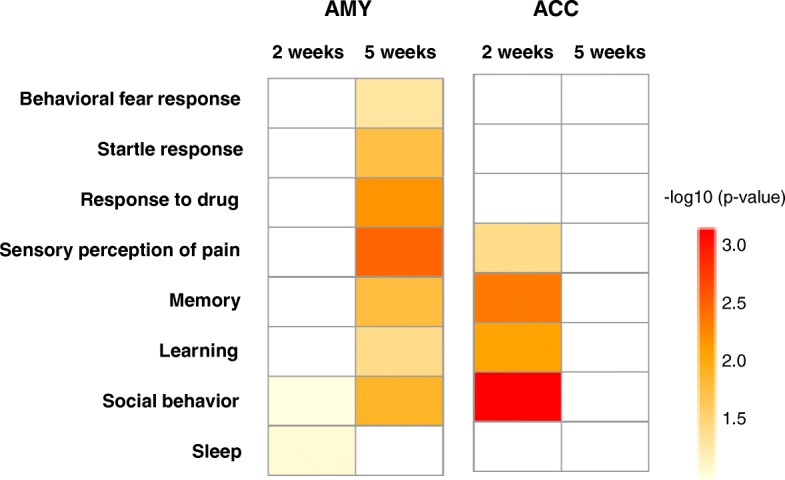
GOs related to PTSD-like behavioral traits. Several GOs in AMY (left columns) and ACC (right columns) were associated with behavioral and cognitive abnormality in PTSD, including fear memory, startle response, drug comorbidity, pain sensitivity, memory and learning, social interaction, and sleep. Color index represents level of significance (*p*-values)

GOs associated with PTSD-like behavior such as fear response, startle response, social behavior were predominantly seen after 5 weeks in AMY and 2 weeks in ACC (Fig. 11). In AMY at 5 weeks PS, Behavioral fear response did not manifest a strong enrichment, though Startle response, Response to drug, Sensory perception of pain, Memory, Learning, and Social behavior were significantly enriched. Relating to the Response to drugs, several GOs were selected including Response to cocaine, amphetamine, morphine, nicotine and ethanol (data not shown). On the other hand, Social behavior and Sleep were listed in 2 weeks PS, despite with a weak enrichment (*p* > 0.05). In ACC at 2 weeks PS, Sensory perception of pain, Social behavior, Memory, and Learning were enriched. Overall, GOs associated with PTSD-like behavior were regulated selectively in AMY at 5 weeks PS and in ACC at 2 weeks PS.

## Discussion

### Behavioral characterization of the foot shock induced stress model

Electric foot shock has been widely used as fear conditioning, because it can provide consistent stress to animals and is easy to assess fear memory. Severe foot shock has also been used as a stressor for PTSD-like animal model [39]. In previous reports, animals receiving electric foot shock was subsequently re-exposed to the same contextual environment [40]. This procedure which is called situational reminder seems critical to acquire long-term PTSD-like abnormal behaviors including long-lasting hyperarousal [26], sustained freezing behavior [25] as well as higher evoked field potential in basolateral AMY [41]. In this study, animals were given the weekly avoidance test and we observed that, except for the first week, all the stressed animals avoid to enter the previously foot-shock chamber, suggesting these stressed animals were threatened under this situation. Together with freezing test at 5 weeks PS, we found that the stressed animals acquired sustained fear memory for more than 1 month. Since anxiety-like behavior is a prominent characteristic of PTSD, we carried out three different behavioral tests (Figs. 3, 4, and 5) and found that the stressed mice acquired anxiety-like behavior, which emerged after 2 weeks and sustained until 4 weeks PS. In contrast, exaggerated acoustic startling response of the stressed mice was only acquired after 4 weeks PS (Fig. 6). Thus, two abnormal behavioral traits in our model were differentially evolved. Moreover, short immobile episodes were more frequent in stressed mice than control animals, suggesting a sleep disturbance in the stressed animals and a reminiscence of the abnormal sleep-wake cycle in PTSD patients.

Differential susceptibility of animals to stress has been recognized as an important trait of the animal model because of the existence of human resilience or vulnerability to trauma [42, 43]. In fact, we observed a wider sample variance in stressed group than in control group in the acoustic startle test at 4 weeks PS (Fig. 6c and e), but not at 2 weeks PS (Fig. 6b and d), suggesting that differential susceptibility was created when stress induced hyperarousal was prominent. Cohen’s group has evaluated individual rodent susceptibility based on the acoustic startle response [44]. Currently it is unknown whether the wide variation was derived from individual difference occurred during the development of PTSD-like abnormality, since there is lack of consistency for the stressed animals to behave as vulnerable or resilient in different behavioral assays.

### Gene regulation associated with PTSD

Genome-wide association studies of human PTSD have identified the disease specific polymorphism of several associated genes in their gene loci. Some of the associated genes are involved in HPA axis and glucocorticoids to regulate stress response and emotional control [13]. In our model, several genes were regulated in either AMY or ACC, including *Crhr1* and *2* [45–47]*, Rora* [48], *Sgk1* [49], and *Stat5b* [50] (Table 1). Among them, FKBP5 has been intensively investigated since it is a co-chaperone for glucocorticoid (GC) receptor to suppress its transcriptional activity [51]. In addition, we found genes involved in synaptic plasticity: *Adcy8*, whose deficiency led to stress-induced anxiety [52] implying a role in long-term potentiation and synaptic plasticity for fear related memory [38], *Cacna1c* (Cav1.2 subunit of L-type calcium channel), which mediates long-term synaptic plasticity through calcium signaling, *Klhl1*, the actin binding protein modulating calcium current [53] and neurite outgrowth [54], and *Shank1*, the anchoring protein playing a role in glutamatergic synaptic plasticity [55]. These results suggest that those PTSD-associated genes can play an important role on the disease development.

### Time-dependent gene regulation in AMY

Numerical gene regulation was comparable between 2 weeks and 5 weeks PS since there were more than 1000 DEGs in each condition (Additional file 2: Figure S2). However, DEGs in the overlapped pathways were predominantly in either 2 weeks or 5 weeks PS, but only a few DEGs overlapped, consistent with the transcription related DEGs (Table 2). Alteration of drastic transcriptional regulatory genes resulted in dynamic changes of global gene expression including synapse remodeling or hormone/neuroendocrine system. In addition, histone mediated epigenetic regulation may contribute to time-dependent regulation (Additional file 5: Figure S3), as recently postulated that epigenetic alteration is deeply involved in the pathogenesis as well as in the disease susceptibility [43, 56, 57]. Of note, the glutamatergic, GABAergic and endocannabinoid signaling were all highly enriched in 5 weeks PS (Fig. 8), consistent with the notion that the excitatory and inhibitory neurotransmission is tightly regulated by the endocannabinoid signaling under stressful conditions [58]. In addition, cholinergic and serotonergic input can modulate neurotransmission at 5 weeks PS. Ras, Rap1, and MAPK pathways which play an important role in synaptic transmission, including fear memory encoding and expression [59, 60] and calcium mediated signaling, including LTP were also predominantly regulated (Fig. 8). Moreover, hormone and neuroendocrine system were also predominantly enriched in 5 weeks PS (Fig. 9). However, we did not find significantly regulated expression of the core HPA axis genes including corticotropin releasing hormone, its receptor, or GC receptor, which was different from a previous study [61]. It is possible that the differences might be derived from the different animal species or experimental procedures. PTSD-like model constructed by foot shock followed by situational reminder may not clearly provoke HPA axis activation in male animal as reported previously [62]. Instead, we found that oxytocin signaling, which is known to exert anxiolytic and antidepressant effect [63, 64], and also as an important negative modulator of HPA axis and GC signaling [65], was highly enriched in 5 weeks PS (Fig. 9). In fact, oxytocin was upregulated in 2 weeks PS, then plummeted in 5 weeks PS. Oxytocin receptor gene was also upregulated in 2 weeks PS, while returned to basal level in 5 weeks PS. Thus, there is a possibility that extra-hypothalamic GC signaling is modulated by regulatory neuroendocrine system such as oxytocin, although further investigation is necessary to clarify the regulation of HPA axis. Moreover, several GOs that are related to the PTSD-like behavioral traits such as startle response, response to drug, and sensory perception to pain were enriched at 5 weeks PS (Fig. 11), suggesting that those DEGs play a key role in the molecular mechanism to disease development. Taken together, gene regulation involved in synapse remodeling and hormone/neuroendocrine system in AMY is likely culminated in 5 weeks PS, which might be correlated to the development of PTSD-like abnormal behaviors.

### Termination of gene regulation after 5 weeks in ACC

ACC is reported to alter the neuronal activity in PTSD patients during fear conditioning test as well as in the resting condition [66]. ACC has extensive afferent connections to emotion regulatory limbic regions including AMY and is involved in emotional learning and certain social behavior [67]. Given its proximity and connection to the limbic structures, ACC is thought to be the direct top-down regulator of PTSD susceptibility through modulating AMY activation to threatening stimuli [7, 8]. Despite that the volume of ACC is reduced in PTSD, some studies have shown hyperactivation of the ACC rather than hypoactivation [10]. Thus, regulation of AMY activation as well as PTSD development by ACC remains to be enigmatic. Our gene analyses in the region found that more than 3000 genes were regulated at 2 weeks PS, then plunged to 43 DEGs at 5 weeks PS, which suggests that gene regulation was mostly terminated at 5 weeks PS (Fig. 6). In addition to synapse remodeling and neuroendocrine and hormone pathways, 18 DEGs related to oxidative phosphorylation were all upregulated specifically in 2 weeks PS (data not shown), suggesting that neuronal activity as well as gene regulation was accelerated together with enhanced mitochondrial activity. Furthermore, a possible anxiogenic gene *Crhr1* [47] was up-regulated whereas the anxiolytic gene *Crhr2* [45, 46] was down-regulated (Table 1), implying the anxiogenic state at 2 weeks PS.

To date, genome-wide transcriptomic studies for PTSD-like model animals have been performed by several groups [68–70]. Muhie et al. reported using aggressor exposed animal models that genes involved in synaptic plasticity, neurogenesis, inflammation, obesity and cardiac infarction pathways were differentially regulated [71]. Even their model construction and sampling procedures were different, enriched KEGG pathways were remarkably similar to our results, especially for synaptic plasticity and neuroendocrine systems. On the other hand, human genomic analyses have identified genes with single nucleotide mutations associated with PTSD. Thus, the results generated from our PTSD-like mouse model constructed by a combination of inescapable foot shock (physical stressor) and repeated situational reminders (psychological stressor) is consistent with the previous reports from other laboratories. Nonetheless, we are aware that similar to the other animal models of human diseases, our current model system may only represent certain aspects of PTSD symptoms. We have not completely characterized our model animals to fulfill face validity, for example the Sucrose preference test to assess anhedonia, Social interaction test for social numbness, and Morris water maze test for memory impairment. Despite the limitation of the current model system, the RNA-seq analysis can partially reflect the PTSD-specific gene regulation, which is likely to provide a clue for molecular basis of temporal alteration in the brain regions crucial for emotional control and fear memory. This study may also help to identify target genes for intervention at particular brain regions and specific stages during the development of PTSD.

## Additional files

Additional file 1: **Figure S1.** RNAseq analysis in Anterior Cingulate Cortex (ACC) of stressed mice. Total RNA was isolated from ACC by punching brain slice and applied for RNA sequence. Heatmaps of DEGs were shown for 2 weeks (A) and 5 weeks (B) PS. Green bars and orange bars indicated control and stressed animals, respectively. Venn diagram of overlapped DEGs between the two time points was shown in (C). (PPTX 496 kb)

Additional file 2: **Figure S2.** RNAseq analysis in Amygdala (AMY) of stressed mice. Total RNA was isolated from AMY by punching brain slice and applied for RNA sequence. Heatmaps of DEGs were shown for 2 weeks (A) and 5 weeks (B) PS. Green bars and orange bars indicated control and stressed animals, respectively. Venn diagram between the two conditions was shown in (C). (PPTX 491 kb)

Additional file 3: **Table S1.** Enriched molecular functions of regulated genes in AMY and ACC at 2 and 5 weeks post stress. (PPTX 44 kb)

Additional file 4: **Table S2.** Top 20 enriched biological processes of regulated genes in AMY and ACC at 2 and 5 weeks post stress. (PPTX 47 kb)

Additional file 5: **Figure S3.** Heatmap of enriched GOs related to transcriptional regulation. Color index represents level of significance (*p*-values). (PPTX 54 kb)

Additional file 6: **Figure S4.** Heatmap of enriched GOs involved in neuronal development, neurogenesis, and differentiation, and non-neuronal cell differentiation. Color index represents level of significance (*p*-values). (PPTX 53 kb)

Additional file 7: **Table S3.** Enriched pathways of regulated genes in AMY and ACC at 2 and 5 weeks post stress. (PPTX 45 kb)

## Abbreviations

ACC: Anterior cingulate cortex
AMY: Amygdala
CNS: Central nervous system
DAVID: Database for Annotation, Visualization and Integrated Discovery
DEG: Differentially expressed genes
GO: Gene ontology
GR: Glucocorticoid receptor
HPA: Hypothalamic-pituitary-adrenal
PFC: Prefrontal cortex
PS: Post stress
PTSD: Post-traumatic stress disorder
