# Correction to: Region- and time-dependent gene regulation in the amygdala and anterior cingulate cortex of a PTSD-like mouse model

**DOI:** 10.1186/s13041-019-0470-3

**Published:** 2019-05-09

**Authors:** Mikiei Tanaka, Hongyun Li, Xijun Zhang, Jatinder Singh, Clifton L. Dalgard, Matthew Wilkerson, Yumin Zhang

**Affiliations:** 1 Department of Anatomy, Physiology and Genetics, Uniformed Services University of Health Sciences, 4301 Jones Bridge Rd, Bethesda, MD 20814 USA; 2 Collaborative Health Initiative Research Program (CHIRP), Uniformed Services University of Health Sciences, 4301 Jones Bridge Rd, Bethesda, MD 20814 USA

**Correction to: Mol Brain 2019;12:25**

**https://doi.org/10.1186/s13041-019-0449-0**

Following publication of the original article [1], the authors reported that one of the authors’ names was spelled incorrectly. In this Correction the incorrect and correct author name are shown. The original publication of this article has been corrected.

Originally the author name was published as:

- Clifton Dalgard

The correct author name is:

- Clifton L. Dalgard
