# Supplementary figures and images for: Region- and time-dependent gene regulation in the amygdala and anterior cingulate cortex of a PTSD-like mouse model
Source: Mol Brain. 2019 Mar 28;12:25. doi: 10.1186/s13041-019-0449-0 (PMC6438009; doi:10.1186/s13041-019-0449-0)

## Slide 1
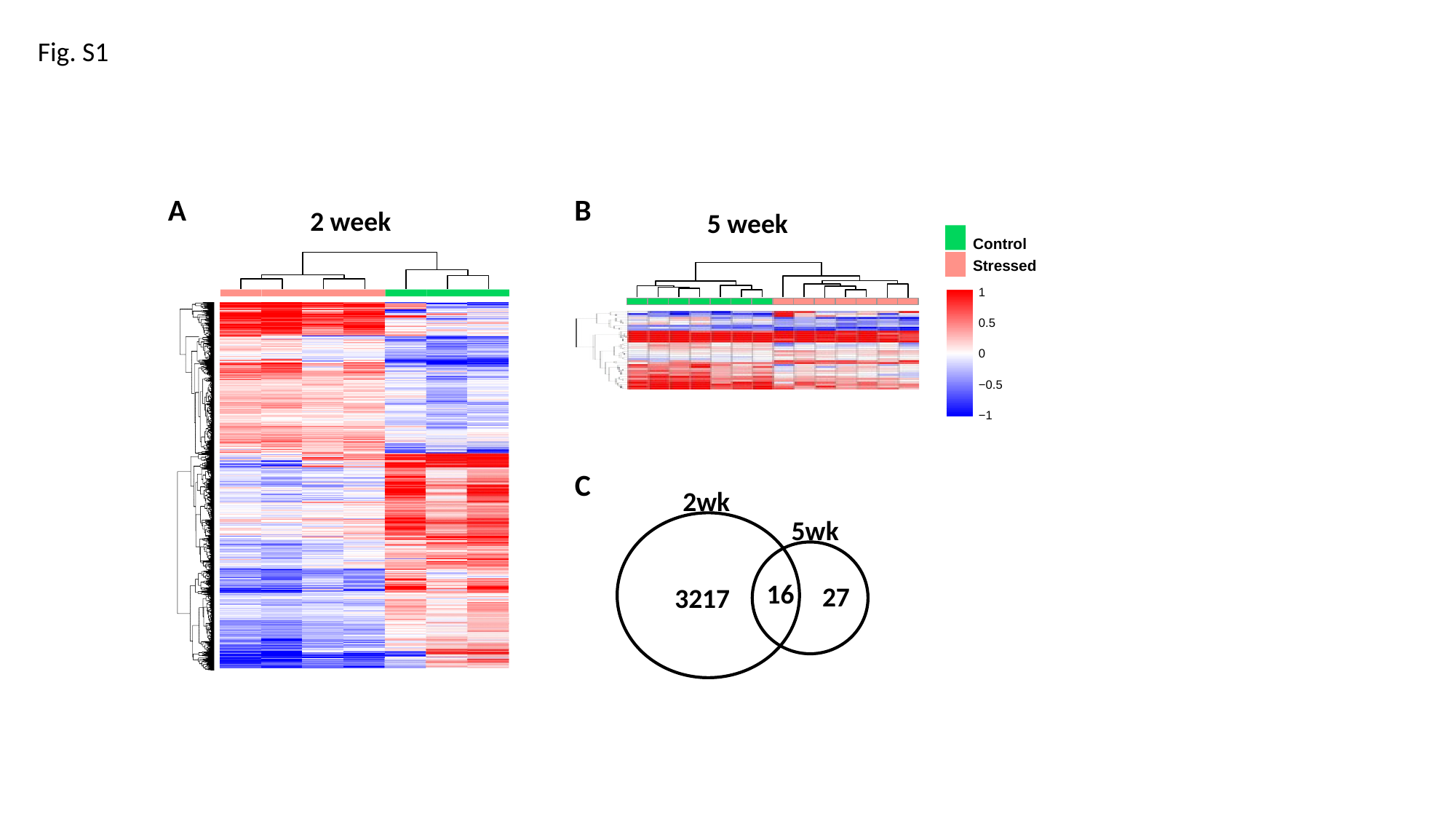

Fig. S1
A
B
2 week
5 week
Control
Stressed
1
0.5
0
−0.5
−1
C
2wk
5wk
16
27
3217

Supplement: Supplementary file 1 — Figure S1. RNAseq analysis in Anterior Cingulate Cortex (ACC) of stressed mice. Total RNA was isolated from ACC by punching brain slice and applied for RNA sequence. Heatmaps of DEGs were shown for 2 weeks (A) and 5 weeks (B) PS. Green bars and orange bars indicated control and stressed animals, respectively. Venn diagram of overlapped DEGs between the two time points was shown in (C). (PPTX 496 kb) [file 13041_2019_449_MOESM1_ESM.pptx]

## Slide 1
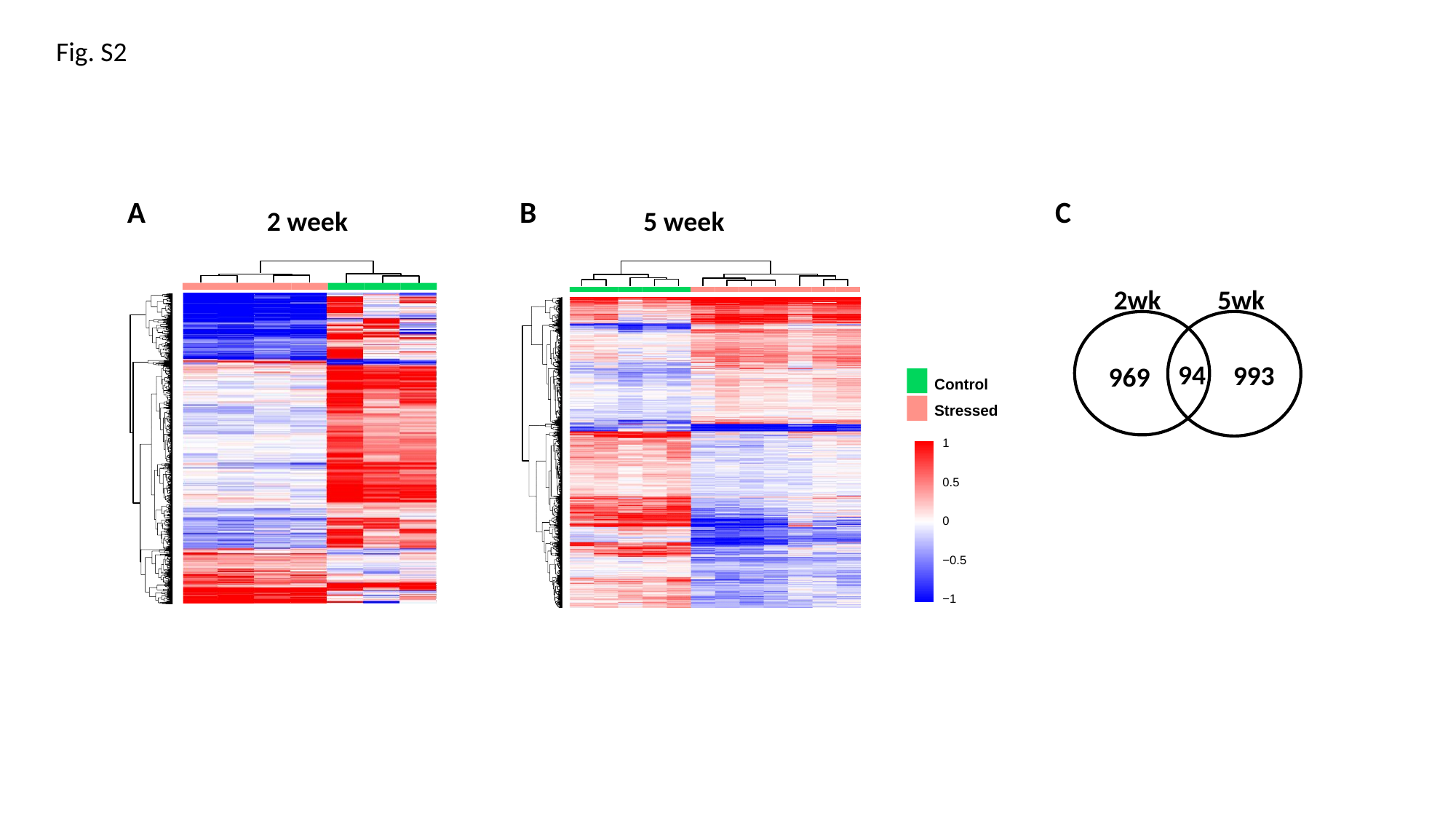

Fig. S2
A
B
C
2 week
5 week
2wk
5wk
94
993
969
Control
Stressed
1
0.5
0
−0.5
−1

Supplement: Supplementary file 2 — Figure S2. RNAseq analysis in Amygdala (AMY) of stressed mice. Total RNA was isolated from AMY by punching brain slice and applied for RNA sequence. Heatmaps of DEGs were shown for 2 weeks (A) and 5 weeks (B) PS. Green bars and orange bars indicated control and stressed animals, respectively. Venn diagram between the two conditions was shown in (C). (PPTX 491 kb) [file 13041_2019_449_MOESM2_ESM.pptx]
