# Supplementary material for: Region- and time-dependent gene regulation in the amygdala and anterior cingulate cortex of a PTSD-like mouse model
Source: Mol Brain. 2019 Mar 28;12:25. doi: 10.1186/s13041-019-0449-0 (PMC6438009; doi:10.1186/s13041-019-0449-0)
Supplement: Supplementary file 3 — Table S1. Enriched molecular functions of regulated genes in AMY and ACC at 2 and 5 weeks post stress. (PPTX 44 kb) [file 13041_2019_449_MOESM3_ESM.pptx]

## Slide 1
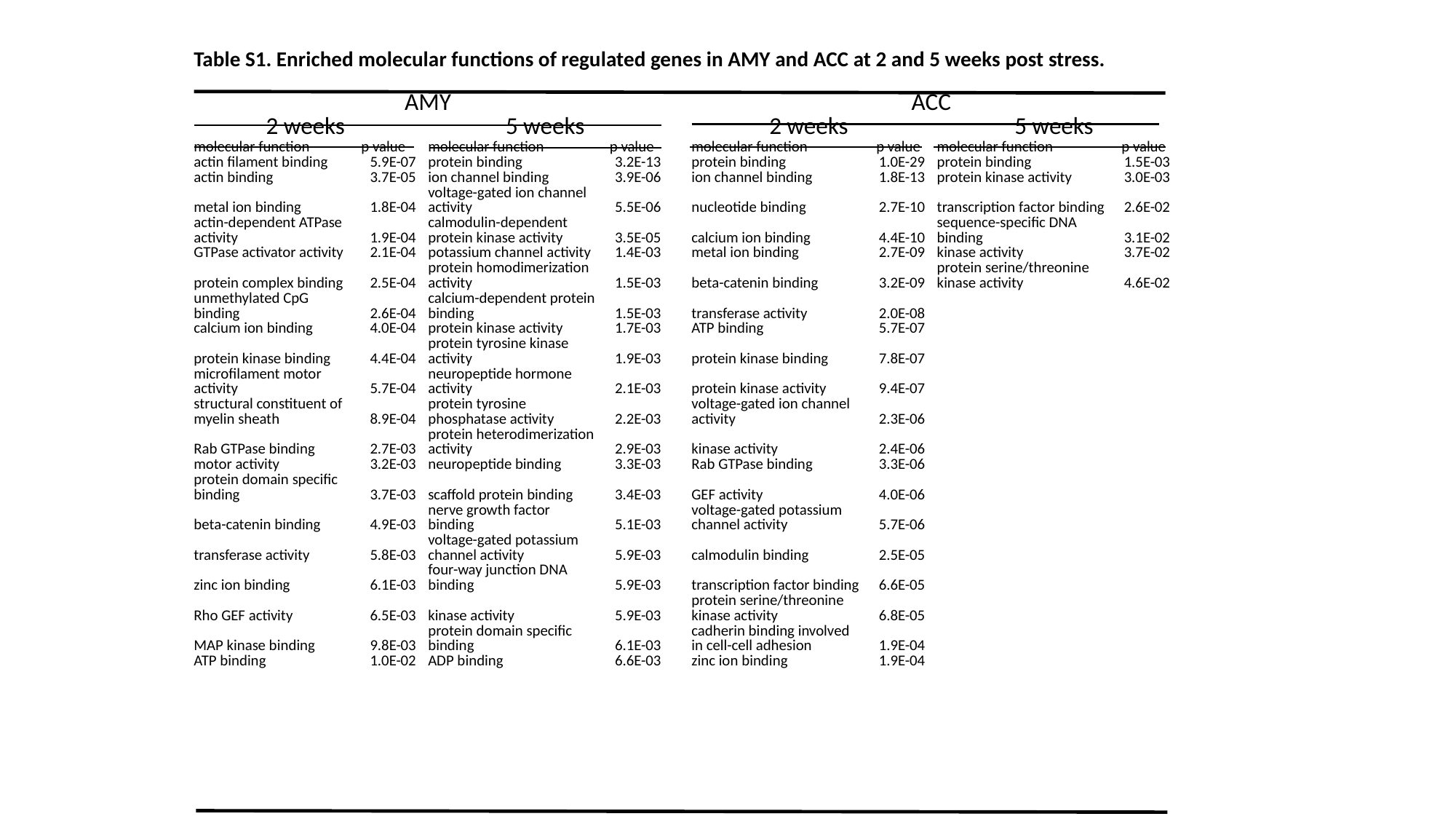

Table S1. Enriched molecular functions of regulated genes in AMY and ACC at 2 and 5 weeks post stress.
| AMY | | | | | ACC | | | |
| --- | --- | --- | --- | --- | --- | --- | --- | --- |
| 2 weeks | | 5 weeks | | | 2 weeks | | 5 weeks | |
| molecular function | p value | molecular function | p value | | molecular function | p value | molecular function | p value |
| actin filament binding | 5.9E-07 | protein binding | 3.2E-13 | | protein binding | 1.0E-29 | protein binding | 1.5E-03 |
| actin binding | 3.7E-05 | ion channel binding | 3.9E-06 | | ion channel binding | 1.8E-13 | protein kinase activity | 3.0E-03 |
| metal ion binding | 1.8E-04 | voltage-gated ion channel activity | 5.5E-06 | | nucleotide binding | 2.7E-10 | transcription factor binding | 2.6E-02 |
| actin-dependent ATPase activity | 1.9E-04 | calmodulin-dependent protein kinase activity | 3.5E-05 | | calcium ion binding | 4.4E-10 | sequence-specific DNA binding | 3.1E-02 |
| GTPase activator activity | 2.1E-04 | potassium channel activity | 1.4E-03 | | metal ion binding | 2.7E-09 | kinase activity | 3.7E-02 |
| protein complex binding | 2.5E-04 | protein homodimerization activity | 1.5E-03 | | beta-catenin binding | 3.2E-09 | protein serine/threonine kinase activity | 4.6E-02 |
| unmethylated CpG binding | 2.6E-04 | calcium-dependent protein binding | 1.5E-03 | | transferase activity | 2.0E-08 | | |
| calcium ion binding | 4.0E-04 | protein kinase activity | 1.7E-03 | | ATP binding | 5.7E-07 | | |
| protein kinase binding | 4.4E-04 | protein tyrosine kinase activity | 1.9E-03 | | protein kinase binding | 7.8E-07 | | |
| microfilament motor activity | 5.7E-04 | neuropeptide hormone activity | 2.1E-03 | | protein kinase activity | 9.4E-07 | | |
| structural constituent of myelin sheath | 8.9E-04 | protein tyrosine phosphatase activity | 2.2E-03 | | voltage-gated ion channel activity | 2.3E-06 | | |
| Rab GTPase binding | 2.7E-03 | protein heterodimerization activity | 2.9E-03 | | kinase activity | 2.4E-06 | | |
| motor activity | 3.2E-03 | neuropeptide binding | 3.3E-03 | | Rab GTPase binding | 3.3E-06 | | |
| protein domain specific binding | 3.7E-03 | scaffold protein binding | 3.4E-03 | | GEF activity | 4.0E-06 | | |
| beta-catenin binding | 4.9E-03 | nerve growth factor binding | 5.1E-03 | | voltage-gated potassium channel activity | 5.7E-06 | | |
| transferase activity | 5.8E-03 | voltage-gated potassium channel activity | 5.9E-03 | | calmodulin binding | 2.5E-05 | | |
| zinc ion binding | 6.1E-03 | four-way junction DNA binding | 5.9E-03 | | transcription factor binding | 6.6E-05 | | |
| Rho GEF activity | 6.5E-03 | kinase activity | 5.9E-03 | | protein serine/threonine kinase activity | 6.8E-05 | | |
| MAP kinase binding | 9.8E-03 | protein domain specific binding | 6.1E-03 | | cadherin binding involved in cell-cell adhesion | 1.9E-04 | | |
| ATP binding | 1.0E-02 | ADP binding | 6.6E-03 | | zinc ion binding | 1.9E-04 | | |
