# Supplementary material for: Region- and time-dependent gene regulation in the amygdala and anterior cingulate cortex of a PTSD-like mouse model
Source: Mol Brain. 2019 Mar 28;12:25. doi: 10.1186/s13041-019-0449-0 (PMC6438009; doi:10.1186/s13041-019-0449-0)
Supplement: Supplementary file 4 — Table S2. Top 20 enriched biological processes of regulated genes in AMY and ACC at 2 and 5 weeks post stress. (PPTX 47 kb) [file 13041_2019_449_MOESM4_ESM.pptx]

## Slide 1
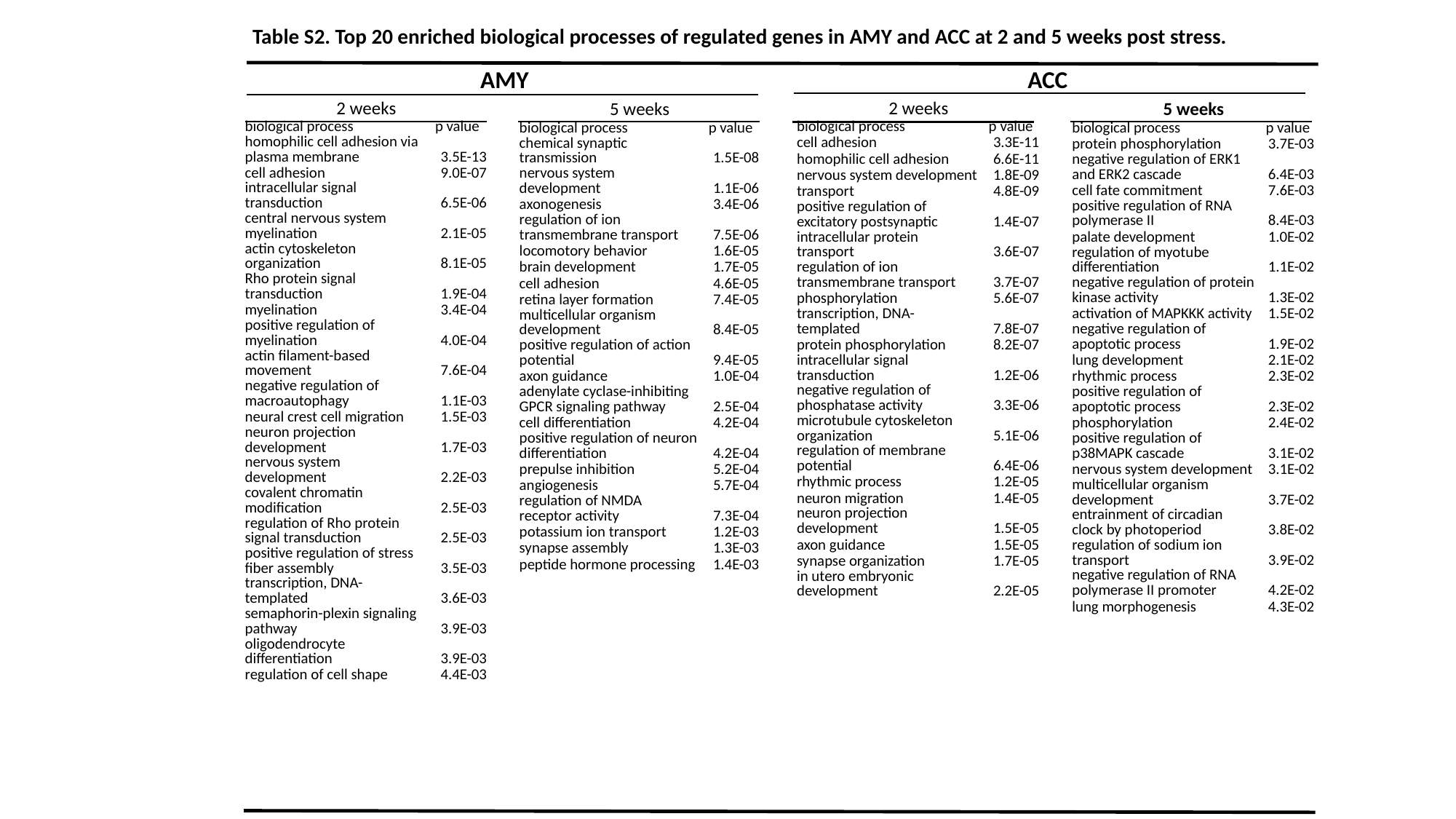

Table S2. Top 20 enriched biological processes of regulated genes in AMY and ACC at 2 and 5 weeks post stress.
AMY
ACC
| 2 weeks | |
| --- | --- |
| biological process | p value |
| homophilic cell adhesion via plasma membrane | 3.5E-13 |
| cell adhesion | 9.0E-07 |
| intracellular signal transduction | 6.5E-06 |
| central nervous system myelination | 2.1E-05 |
| actin cytoskeleton organization | 8.1E-05 |
| Rho protein signal transduction | 1.9E-04 |
| myelination | 3.4E-04 |
| positive regulation of myelination | 4.0E-04 |
| actin filament-based movement | 7.6E-04 |
| negative regulation of macroautophagy | 1.1E-03 |
| neural crest cell migration | 1.5E-03 |
| neuron projection development | 1.7E-03 |
| nervous system development | 2.2E-03 |
| covalent chromatin modification | 2.5E-03 |
| regulation of Rho protein signal transduction | 2.5E-03 |
| positive regulation of stress fiber assembly | 3.5E-03 |
| transcription, DNA-templated | 3.6E-03 |
| semaphorin-plexin signaling pathway | 3.9E-03 |
| oligodendrocyte differentiation | 3.9E-03 |
| regulation of cell shape | 4.4E-03 |
| 2 weeks | |
| --- | --- |
| biological process | p value |
| cell adhesion | 3.3E-11 |
| homophilic cell adhesion | 6.6E-11 |
| nervous system development | 1.8E-09 |
| transport | 4.8E-09 |
| positive regulation of excitatory postsynaptic | 1.4E-07 |
| intracellular protein transport | 3.6E-07 |
| regulation of ion transmembrane transport | 3.7E-07 |
| phosphorylation | 5.6E-07 |
| transcription, DNA-templated | 7.8E-07 |
| protein phosphorylation | 8.2E-07 |
| intracellular signal transduction | 1.2E-06 |
| negative regulation of phosphatase activity | 3.3E-06 |
| microtubule cytoskeleton organization | 5.1E-06 |
| regulation of membrane potential | 6.4E-06 |
| rhythmic process | 1.2E-05 |
| neuron migration | 1.4E-05 |
| neuron projection development | 1.5E-05 |
| axon guidance | 1.5E-05 |
| synapse organization | 1.7E-05 |
| in utero embryonic development | 2.2E-05 |
| 5 weeks | |
| --- | --- |
| biological process | p value |
| chemical synaptic transmission | 1.5E-08 |
| nervous system development | 1.1E-06 |
| axonogenesis | 3.4E-06 |
| regulation of ion transmembrane transport | 7.5E-06 |
| locomotory behavior | 1.6E-05 |
| brain development | 1.7E-05 |
| cell adhesion | 4.6E-05 |
| retina layer formation | 7.4E-05 |
| multicellular organism development | 8.4E-05 |
| positive regulation of action potential | 9.4E-05 |
| axon guidance | 1.0E-04 |
| adenylate cyclase-inhibiting GPCR signaling pathway | 2.5E-04 |
| cell differentiation | 4.2E-04 |
| positive regulation of neuron differentiation | 4.2E-04 |
| prepulse inhibition | 5.2E-04 |
| angiogenesis | 5.7E-04 |
| regulation of NMDA receptor activity | 7.3E-04 |
| potassium ion transport | 1.2E-03 |
| synapse assembly | 1.3E-03 |
| peptide hormone processing | 1.4E-03 |
| 5 weeks | |
| --- | --- |
| biological process | p value |
| protein phosphorylation | 3.7E-03 |
| negative regulation of ERK1 and ERK2 cascade | 6.4E-03 |
| cell fate commitment | 7.6E-03 |
| positive regulation of RNA polymerase II | 8.4E-03 |
| palate development | 1.0E-02 |
| regulation of myotube differentiation | 1.1E-02 |
| negative regulation of protein kinase activity | 1.3E-02 |
| activation of MAPKKK activity | 1.5E-02 |
| negative regulation of apoptotic process | 1.9E-02 |
| lung development | 2.1E-02 |
| rhythmic process | 2.3E-02 |
| positive regulation of apoptotic process | 2.3E-02 |
| phosphorylation | 2.4E-02 |
| positive regulation of p38MAPK cascade | 3.1E-02 |
| nervous system development | 3.1E-02 |
| multicellular organism development | 3.7E-02 |
| entrainment of circadian clock by photoperiod | 3.8E-02 |
| regulation of sodium ion transport | 3.9E-02 |
| negative regulation of RNA polymerase II promoter | 4.2E-02 |
| lung morphogenesis | 4.3E-02 |
