# Supplementary material for: Region- and time-dependent gene regulation in the amygdala and anterior cingulate cortex of a PTSD-like mouse model
Source: Mol Brain. 2019 Mar 28;12:25. doi: 10.1186/s13041-019-0449-0 (PMC6438009; doi:10.1186/s13041-019-0449-0)
Supplement: Supplementary file 5 — Figure S3. Heatmap of enriched GOs related to transcriptional regulation. Color index represents level of significance (p-values). (PPTX 54 kb) [file 13041_2019_449_MOESM5_ESM.pptx]

## Slide 1
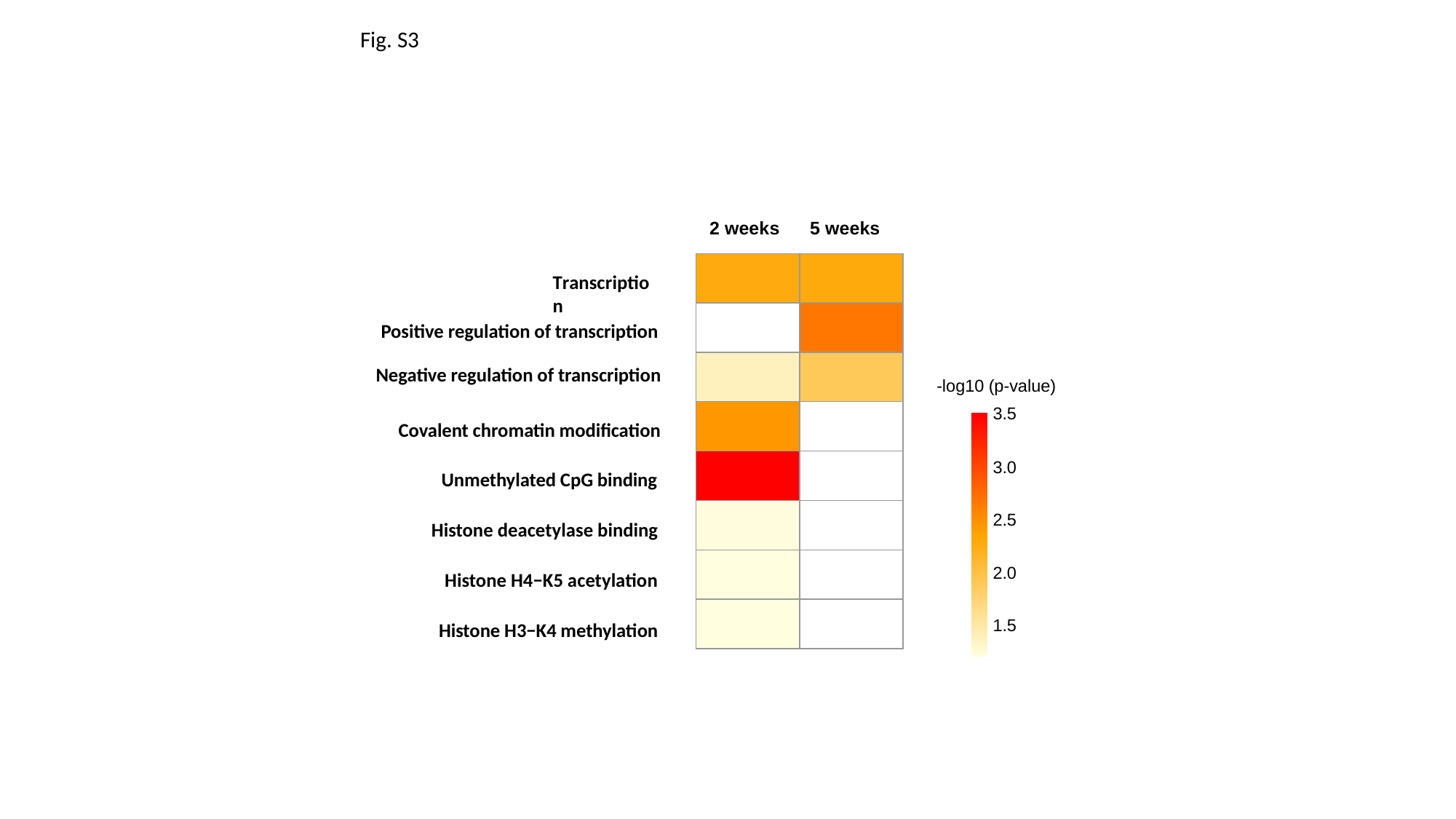

Fig. S3
2 weeks 5 weeks
| | |
| --- | --- |
| | |
| | |
| | |
| | |
| | |
| | |
| | |
Transcription
Positive regulation of transcription
Negative regulation of transcription
-log10 (p-value)
3.5
Covalent chromatin modification
3.0
Unmethylated CpG binding
2.5
Histone deacetylase binding
2.0
Histone H4−K5 acetylation
1.5
Histone H3−K4 methylation
