# Supplementary material for: Region- and time-dependent gene regulation in the amygdala and anterior cingulate cortex of a PTSD-like mouse model
Source: Mol Brain. 2019 Mar 28;12:25. doi: 10.1186/s13041-019-0449-0 (PMC6438009; doi:10.1186/s13041-019-0449-0)
Supplement: Supplementary file 6 — Figure S4. Heatmap of enriched GOs involved in neuronal development, neurogenesis, and differentiation, and non-neuronal cell differentiation. Color index represents level of significance (p-values). (PPTX 53 kb) [file 13041_2019_449_MOESM6_ESM.pptx]

## Slide 1
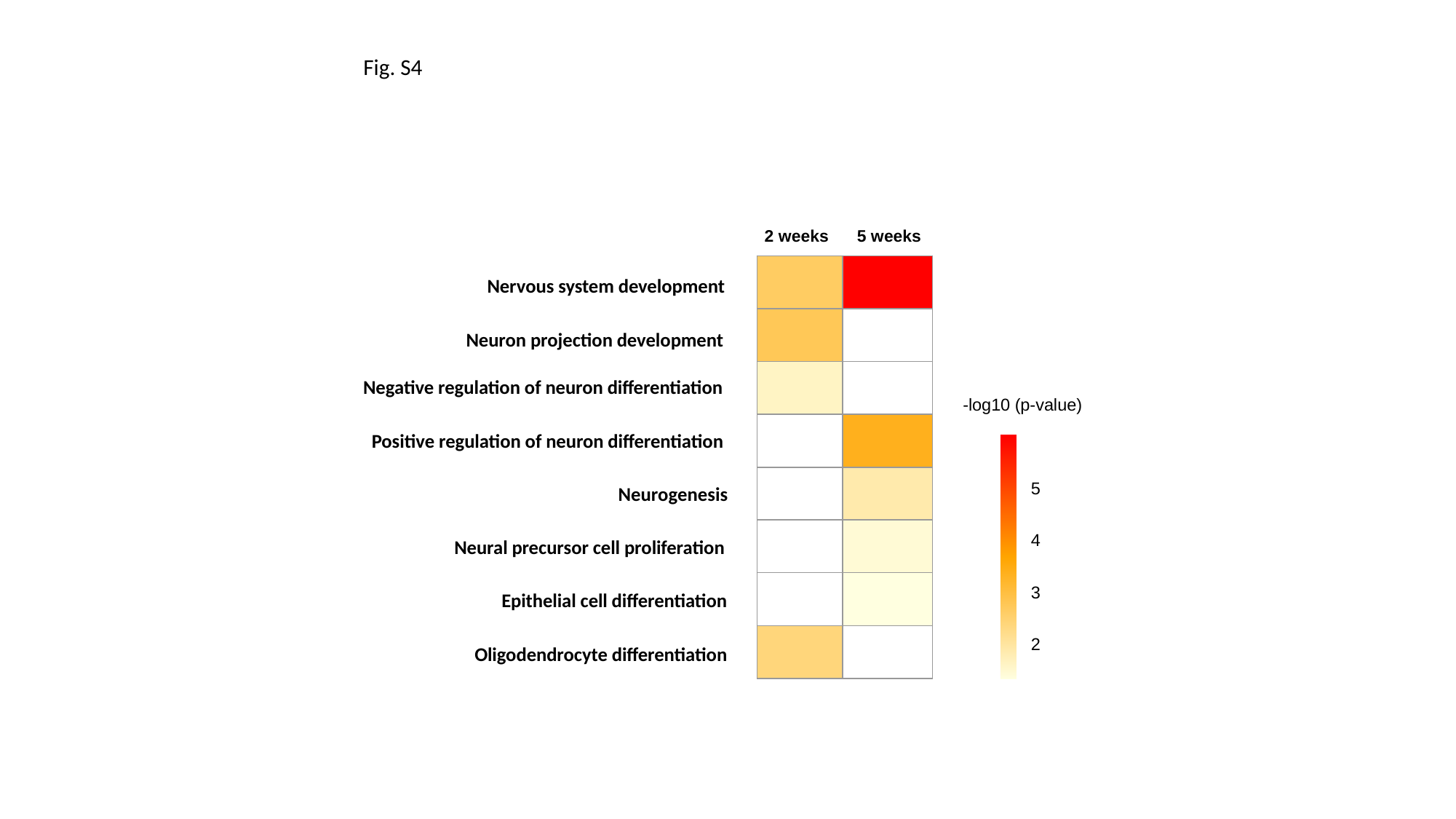

Fig. S4
2 weeks 5 weeks
| | |
| --- | --- |
| | |
| | |
| | |
| | |
| | |
| | |
| | |
Nervous system development
Neuron projection development
Negative regulation of neuron differentiation
-log10 (p-value)
Positive regulation of neuron differentiation
5
4
3
2
Neurogenesis
Neural precursor cell proliferation
Epithelial cell differentiation
Oligodendrocyte differentiation
