# Supplementary material for: Region- and time-dependent gene regulation in the amygdala and anterior cingulate cortex of a PTSD-like mouse model
Source: Mol Brain. 2019 Mar 28;12:25. doi: 10.1186/s13041-019-0449-0 (PMC6438009; doi:10.1186/s13041-019-0449-0)
Supplement: Supplementary file 7 — Table S3. Enriched pathways of regulated genes in AMY and ACC at 2 and 5 weeks post stress. (PPTX 45 kb) [file 13041_2019_449_MOESM7_ESM.pptx]

## Slide 1
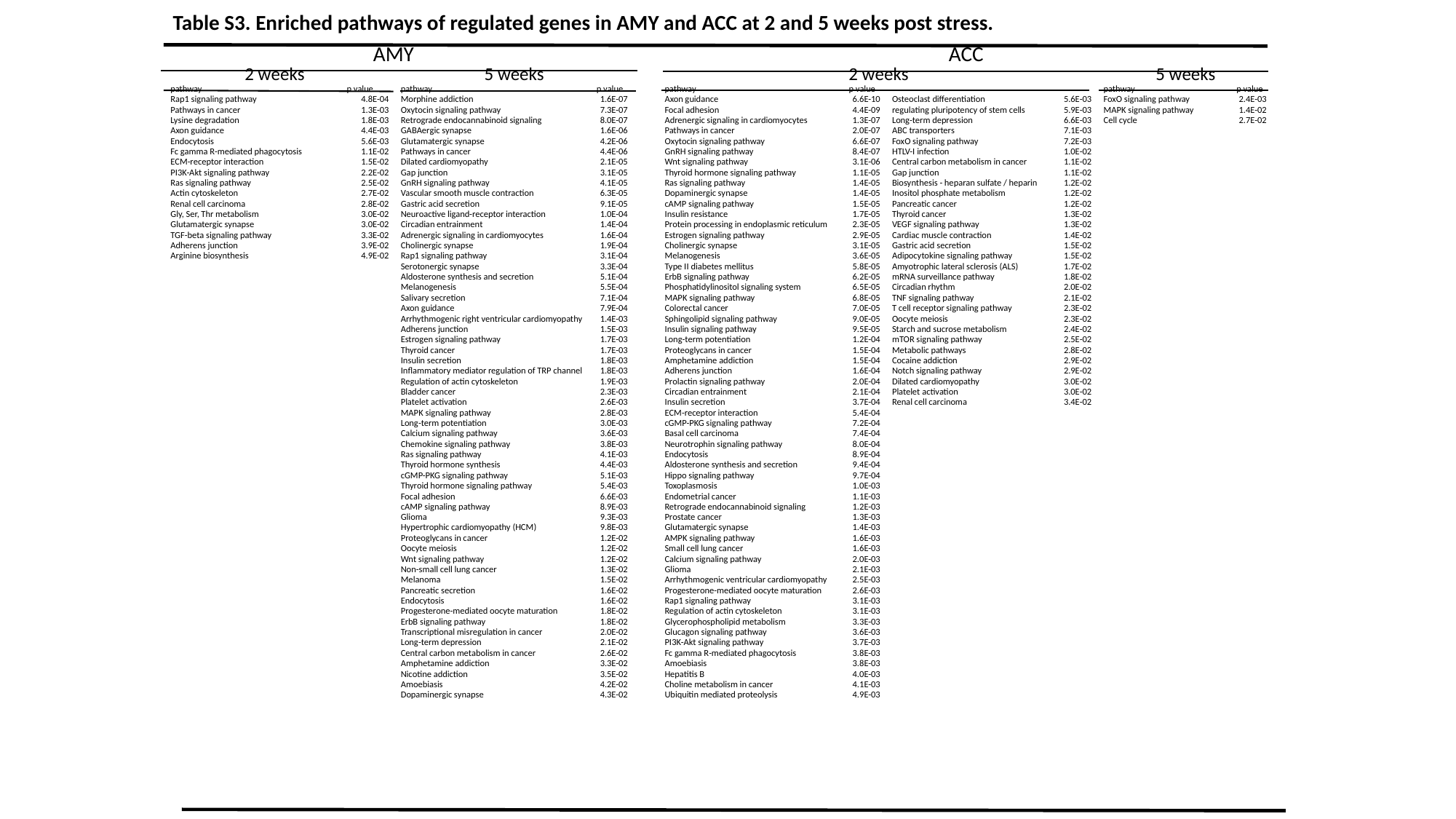

Table S3. Enriched pathways of regulated genes in AMY and ACC at 2 and 5 weeks post stress.
| AMY | | | | | ACC | | | | | |
| --- | --- | --- | --- | --- | --- | --- | --- | --- | --- | --- |
| 2 weeks | | 5 weeks | | | 2 weeks | | | | 5 weeks | |
| pathway | p value | pathway | p value | | pathway | p value | | | pathway | p value |
| Rap1 signaling pathway | 4.8E-04 | Morphine addiction | 1.6E-07 | | Axon guidance | 6.6E-10 | Osteoclast differentiation | 5.6E-03 | FoxO signaling pathway | 2.4E-03 |
| Pathways in cancer | 1.3E-03 | Oxytocin signaling pathway | 7.3E-07 | | Focal adhesion | 4.4E-09 | regulating pluripotency of stem cells | 5.9E-03 | MAPK signaling pathway | 1.4E-02 |
| Lysine degradation | 1.8E-03 | Retrograde endocannabinoid signaling | 8.0E-07 | | Adrenergic signaling in cardiomyocytes | 1.3E-07 | Long-term depression | 6.6E-03 | Cell cycle | 2.7E-02 |
| Axon guidance | 4.4E-03 | GABAergic synapse | 1.6E-06 | | Pathways in cancer | 2.0E-07 | ABC transporters | 7.1E-03 | | |
| Endocytosis | 5.6E-03 | Glutamatergic synapse | 4.2E-06 | | Oxytocin signaling pathway | 6.6E-07 | FoxO signaling pathway | 7.2E-03 | | |
| Fc gamma R-mediated phagocytosis | 1.1E-02 | Pathways in cancer | 4.4E-06 | | GnRH signaling pathway | 8.4E-07 | HTLV-I infection | 1.0E-02 | | |
| ECM-receptor interaction | 1.5E-02 | Dilated cardiomyopathy | 2.1E-05 | | Wnt signaling pathway | 3.1E-06 | Central carbon metabolism in cancer | 1.1E-02 | | |
| PI3K-Akt signaling pathway | 2.2E-02 | Gap junction | 3.1E-05 | | Thyroid hormone signaling pathway | 1.1E-05 | Gap junction | 1.1E-02 | | |
| Ras signaling pathway | 2.5E-02 | GnRH signaling pathway | 4.1E-05 | | Ras signaling pathway | 1.4E-05 | Biosynthesis - heparan sulfate / heparin | 1.2E-02 | | |
| Actin cytoskeleton | 2.7E-02 | Vascular smooth muscle contraction | 6.3E-05 | | Dopaminergic synapse | 1.4E-05 | Inositol phosphate metabolism | 1.2E-02 | | |
| Renal cell carcinoma | 2.8E-02 | Gastric acid secretion | 9.1E-05 | | cAMP signaling pathway | 1.5E-05 | Pancreatic cancer | 1.2E-02 | | |
| Gly, Ser, Thr metabolism | 3.0E-02 | Neuroactive ligand-receptor interaction | 1.0E-04 | | Insulin resistance | 1.7E-05 | Thyroid cancer | 1.3E-02 | | |
| Glutamatergic synapse | 3.0E-02 | Circadian entrainment | 1.4E-04 | | Protein processing in endoplasmic reticulum | 2.3E-05 | VEGF signaling pathway | 1.3E-02 | | |
| TGF-beta signaling pathway | 3.3E-02 | Adrenergic signaling in cardiomyocytes | 1.6E-04 | | Estrogen signaling pathway | 2.9E-05 | Cardiac muscle contraction | 1.4E-02 | | |
| Adherens junction | 3.9E-02 | Cholinergic synapse | 1.9E-04 | | Cholinergic synapse | 3.1E-05 | Gastric acid secretion | 1.5E-02 | | |
| Arginine biosynthesis | 4.9E-02 | Rap1 signaling pathway | 3.1E-04 | | Melanogenesis | 3.6E-05 | Adipocytokine signaling pathway | 1.5E-02 | | |
| | | Serotonergic synapse | 3.3E-04 | | Type II diabetes mellitus | 5.8E-05 | Amyotrophic lateral sclerosis (ALS) | 1.7E-02 | | |
| | | Aldosterone synthesis and secretion | 5.1E-04 | | ErbB signaling pathway | 6.2E-05 | mRNA surveillance pathway | 1.8E-02 | | |
| | | Melanogenesis | 5.5E-04 | | Phosphatidylinositol signaling system | 6.5E-05 | Circadian rhythm | 2.0E-02 | | |
| | | Salivary secretion | 7.1E-04 | | MAPK signaling pathway | 6.8E-05 | TNF signaling pathway | 2.1E-02 | | |
| | | Axon guidance | 7.9E-04 | | Colorectal cancer | 7.0E-05 | T cell receptor signaling pathway | 2.3E-02 | | |
| | | Arrhythmogenic right ventricular cardiomyopathy | 1.4E-03 | | Sphingolipid signaling pathway | 9.0E-05 | Oocyte meiosis | 2.3E-02 | | |
| | | Adherens junction | 1.5E-03 | | Insulin signaling pathway | 9.5E-05 | Starch and sucrose metabolism | 2.4E-02 | | |
| | | Estrogen signaling pathway | 1.7E-03 | | Long-term potentiation | 1.2E-04 | mTOR signaling pathway | 2.5E-02 | | |
| | | Thyroid cancer | 1.7E-03 | | Proteoglycans in cancer | 1.5E-04 | Metabolic pathways | 2.8E-02 | | |
| | | Insulin secretion | 1.8E-03 | | Amphetamine addiction | 1.5E-04 | Cocaine addiction | 2.9E-02 | | |
| | | Inflammatory mediator regulation of TRP channel | 1.8E-03 | | Adherens junction | 1.6E-04 | Notch signaling pathway | 2.9E-02 | | |
| | | Regulation of actin cytoskeleton | 1.9E-03 | | Prolactin signaling pathway | 2.0E-04 | Dilated cardiomyopathy | 3.0E-02 | | |
| | | Bladder cancer | 2.3E-03 | | Circadian entrainment | 2.1E-04 | Platelet activation | 3.0E-02 | | |
| | | Platelet activation | 2.6E-03 | | Insulin secretion | 3.7E-04 | Renal cell carcinoma | 3.4E-02 | | |
| | | MAPK signaling pathway | 2.8E-03 | | ECM-receptor interaction | 5.4E-04 | | | | |
| | | Long-term potentiation | 3.0E-03 | | cGMP-PKG signaling pathway | 7.2E-04 | | | | |
| | | Calcium signaling pathway | 3.6E-03 | | Basal cell carcinoma | 7.4E-04 | | | | |
| | | Chemokine signaling pathway | 3.8E-03 | | Neurotrophin signaling pathway | 8.0E-04 | | | | |
| | | Ras signaling pathway | 4.1E-03 | | Endocytosis | 8.9E-04 | | | | |
| | | Thyroid hormone synthesis | 4.4E-03 | | Aldosterone synthesis and secretion | 9.4E-04 | | | | |
| | | cGMP-PKG signaling pathway | 5.1E-03 | | Hippo signaling pathway | 9.7E-04 | | | | |
| | | Thyroid hormone signaling pathway | 5.4E-03 | | Toxoplasmosis | 1.0E-03 | | | | |
| | | Focal adhesion | 6.6E-03 | | Endometrial cancer | 1.1E-03 | | | | |
| | | cAMP signaling pathway | 8.9E-03 | | Retrograde endocannabinoid signaling | 1.2E-03 | | | | |
| | | Glioma | 9.3E-03 | | Prostate cancer | 1.3E-03 | | | | |
| | | Hypertrophic cardiomyopathy (HCM) | 9.8E-03 | | Glutamatergic synapse | 1.4E-03 | | | | |
| | | Proteoglycans in cancer | 1.2E-02 | | AMPK signaling pathway | 1.6E-03 | | | | |
| | | Oocyte meiosis | 1.2E-02 | | Small cell lung cancer | 1.6E-03 | | | | |
| | | Wnt signaling pathway | 1.2E-02 | | Calcium signaling pathway | 2.0E-03 | | | | |
| | | Non-small cell lung cancer | 1.3E-02 | | Glioma | 2.1E-03 | | | | |
| | | Melanoma | 1.5E-02 | | Arrhythmogenic ventricular cardiomyopathy | 2.5E-03 | | | | |
| | | Pancreatic secretion | 1.6E-02 | | Progesterone-mediated oocyte maturation | 2.6E-03 | | | | |
| | | Endocytosis | 1.6E-02 | | Rap1 signaling pathway | 3.1E-03 | | | | |
| | | Progesterone-mediated oocyte maturation | 1.8E-02 | | Regulation of actin cytoskeleton | 3.1E-03 | | | | |
| | | ErbB signaling pathway | 1.8E-02 | | Glycerophospholipid metabolism | 3.3E-03 | | | | |
| | | Transcriptional misregulation in cancer | 2.0E-02 | | Glucagon signaling pathway | 3.6E-03 | | | | |
| | | Long-term depression | 2.1E-02 | | PI3K-Akt signaling pathway | 3.7E-03 | | | | |
| | | Central carbon metabolism in cancer | 2.6E-02 | | Fc gamma R-mediated phagocytosis | 3.8E-03 | | | | |
| | | Amphetamine addiction | 3.3E-02 | | Amoebiasis | 3.8E-03 | | | | |
| | | Nicotine addiction | 3.5E-02 | | Hepatitis B | 4.0E-03 | | | | |
| | | Amoebiasis | 4.2E-02 | | Choline metabolism in cancer | 4.1E-03 | | | | |
| | | Dopaminergic synapse | 4.3E-02 | | Ubiquitin mediated proteolysis | 4.9E-03 | | | | |
